# UBR5 Acts as an Antiviral Host Factor against MERS-CoV via Promoting Ubiquitination and Degradation of ORF4b

**DOI:** 10.1128/jvi.00741-22

**Published:** 2022-08-18

**Authors:** Yuzheng Zhou, Rong Zheng, Donglan Liu, Sixu Liu, Cyrollah Disoma, Shiqin Li, Yujie Liao, Zongpeng Chen, Ashuai Du, Zijun Dong, Yongxing Zhang, Pinjia Liu, Aroona Razzaq, Dingbin Chen, Xuan Chen, Xiankezi Zhong, Sijie Liu, Siyi Tao, Yuxin Liu, Lunan Xu, Xu Deng, Jiada Li, Taijiao Jiang, Jincun Zhao, Shanni Li, Zanxian Xia

**Affiliations:** a Department of Cell Biology, School of Life Sciences, Central South University, Changsha, China; b State Key Laboratory of Respiratory Disease, National Clinical Research Center for Respiratory Disease, Guangzhou Institute of Respiratory Health, the First Affiliated Hospital of Guangzhou Medical University, Guangzhou, China; c Department of Basic Medicine, School of Medicine, Hunan Normal University, Changsha, China; d Xiangya School of Pharmaceutical Science, Central South University, Changsha, China; e Hunan International Scientific and Technological Cooperation Base of Animal Models for Human Disease, Changsha, China; f Center for Systems Medicine, Institute of Basic Medical Sciences, Chinese Academy of Medical Sciences & Peking Union Medical College, Beijing, China; g Institute of Infectious disease, Guangzhou Eighth People's Hospital of Guangzhou Medical University, Guangzhou, China; h Hunan Key Laboratory of Animal Models for Human Diseases, School of Life Sciences, Central South University, Changsha, China; i Hunan Key Laboratory of Medical Genetics, School of Life Sciences, Central South University, Changsha, China; j Center for Medical Genetics, School of Life Sciences, Central South University, Changsha, China; Loyola University Chicago

**Keywords:** coronavirus, MERS-CoV, accessory protein, IRF3, posttranslational modification, antiviral mechanism

## Abstract

Within the past 2 decades, three highly pathogenic human coronaviruses have emerged, namely, severe acute respiratory syndrome coronavirus (SARS-CoV), Middle East respiratory syndrome coronavirus (MERS-CoV), and severe acute respiratory syndrome coronavirus 2 (SARS-CoV-2). The health threats and economic burden posed by these tremendously severe coronaviruses have paved the way for research on their etiology, pathogenesis, and treatment. Compared to SARS-CoV and SARS-CoV-2, MERS-CoV genome encoded fewer accessory proteins, among which the ORF4b protein had anti-immunity ability in both the cytoplasm and nucleus. Our work for the first time revealed that ORF4b protein was unstable in the host cells and could be degraded by the ubiquitin proteasome system. After extensive screenings, it was found that UBR5 (ubiquitin protein ligase E3 component N-recognin 5), a member of the HECT E3 ubiquitin ligases, specifically regulated the ubiquitination and degradation of ORF4b. Similar to ORF4b, UBR5 can also translocate into the nucleus through its nuclear localization signal, enabling it to regulate ORF4b stability in both the cytoplasm and nucleus. Through further experiments, lysine 36 was identified as the ubiquitination site on the ORF4b protein, and this residue was highly conserved in various MERS-CoV strains isolated from different regions. When UBR5 was knocked down, the ability of ORF4b to suppress innate immunity was enhanced and MERS-CoV replication was stronger. As an anti-MERS-CoV host protein, UBR5 targets and degrades ORF4b protein through the ubiquitin proteasome system, thereby attenuating the anti-immunity ability of ORF4b and ultimately inhibiting MERS-CoV immune escape, which is a novel antagonistic mechanism of the host against MERS-CoV infection.

**IMPORTANCE** ORF4b was an accessory protein unique to MERS-CoV and was not present in SARS-CoV and SARS-CoV-2 which can also cause severe respiratory disease. Moreover, ORF4b inhibited the production of antiviral cytokines in both the cytoplasm and the nucleus, which was likely to be associated with the high lethality of MERS-CoV. However, whether the host proteins regulate the function of ORF4b is unknown. Our study first determined that UBR5, a host E3 ligase, was a potential host anti-MERS-CoV protein that could reduce the protein level of ORF4b and diminish its anti-immunity ability by inducing ubiquitination and degradation. Based on the discovery of ORF4b-UBR5, a critical molecular target, further increasing the degradation of ORF4b caused by UBR5 could provide a new strategy for the clinical development of drugs for MERS-CoV.

## INTRODUCTION

Coronaviruses (CoVs) are so far the largest known RNA viruses, with a genome of approximately 30 kb (1). Seven coronaviruses are known to infect humans, three of which can cause a severe form of pneumonia, including severe acute respiratory syndrome coronavirus (SARS-CoV), Middle East respiratory syndrome coronavirus (MERS-CoV), and severe acute respiratory syndrome coronavirus 2 (SARS-CoV-2) (2). According to the WHO, a total of 2,468 cases and 851 deaths had been reported in 27 countries for MERS-CoV as of 10 March 2022. Even with some underreporting, the mortality rate of MERS-CoV is much higher than those of SARS-CoV and SARS-CoV-2 (3). MERS-CoV is a highly pathogenic coronavirus that was first identified in Saudi Arabia in September 2012 (4–7). Following an outbreak of the virus in the Middle East, infections and transmissions were then reported in Asia, Europe, and North America, particularly in Saudi Arabia, Egypt, and South Korea (8, 9). Although MERS-CoV was considered to be predominantly pandemic in dromedary populations in Africa and the Arabian Peninsula, a considerable pandemic risk exists as zoonotic transmission into human populations is still possible (10, 11). However, relatively few studies have been conducted on the etiological pathways and pathogenesis of MERS-CoV. Therefore, an in-depth study of the mutual antagonism between viruses and their human hosts can provide clinical and social relevance, as this kind of study could identify novel antiviral mechanisms of host resistance to MERS-CoV, thereby paving the way for a potentially useful approach for therapeutic intervention.

The viral proteins encoded by MERS-CoV are divided into three major categories: (i) two large polyproteins, (ii) four structural proteins, and (iii) five accessory proteins (2, 12). Among them, accessory proteins were not involved in viral replication or entry, and they were believed to play crucial roles in viral pathogenicity by regulating virus-host interactions and host immune responses. Deletion of the accessory proteins of MERS-CoV resulted in diminished virulence, a decline in the inhibitory effect on interferon (IFN), and unfavorable immune escape of the virus (13). ORF4a, ORF4b, ORF5, and ORF8b have been reported as MERS-CoV accessory proteins with potential interferon resistance, implying that these proteins were likely to be associated with the high lethality of MERS-CoV (14, 15). Interestingly, among these accessory proteins mentioned previously, only ORF4b had a nuclear localization signal that allowed it to enter the nucleus and exerted its anti-interferon effects in both the cytoplasm and the nucleus (14, 16). This property of ORF4b to inhibit interferon at different subcellular locations has attracted our interest. Thus, we aimed to investigate the unknown characteristic of ORF4b protein as a way to explore the interaction between host and virus.

As one of the five most common posttranslational modifications (PTMs), ubiquitination plays an important role in protein localization, metabolism, activation, and degradation. It is involved in almost all life events, such as cell cycle, differentiation, apoptosis, and signaling (17). The role of ubiquitination in tumorigenesis has already been particularly well studied (18, 19), and the function of ubiquitination in host immunity and viral infection has also drawn more attention in recent years (20–24). In pathways that regulate innate immunity, the activation of many key signaling molecules, such as RIG-I and MAVS, needs to be regulated by ubiquitination (25–27). Correspondingly, some viral proteins can suppress host immunity by deubiquitinating RIG-I, MAVS, or STING or inhibiting their binding to the relevant E3 ligases to attenuate their activation (28–31). They can also use E3 ligases of their own or of the host to specifically degrade host antiviral factors to function as immune escape agents (32–35). Conversely, the host can degrade some viral proteins via the ubiquitin proteasome system, thus weakening the infectivity and pathogenicity of the virus (36–38). Taken together, ubiquitination plays an important role in host immunity and viral pathogenicity and is a double-edged sword for both the host and the virus (39).

Our study is the first to report that ORF4b of MERS-CoV is an unstable protein in host cells. This study showed that polyubiquitination of ORF4b on lysine 36 led to its proteasomal degradation. After screening, we identified UBR5 as the only E3 ligase that specifically executed the polyubiquitination of ORF4b in both the cytoplasm and nucleus, which then abrogated the ability of ORF4b to suppress the host innate immune system. Because of the dose-dependent ability of ORF4b protein to resist host immunity, we proposed a new mechanism of host antagonism against MERS-CoV, highlighting its therapeutic role by suggesting a new antiviral strategy and target for the treatment of MERS-CoV.

## RESULTS

### Identifications of MERS-CoV ORF4b functions and its host’s interacting proteins.

**FIG 1:**
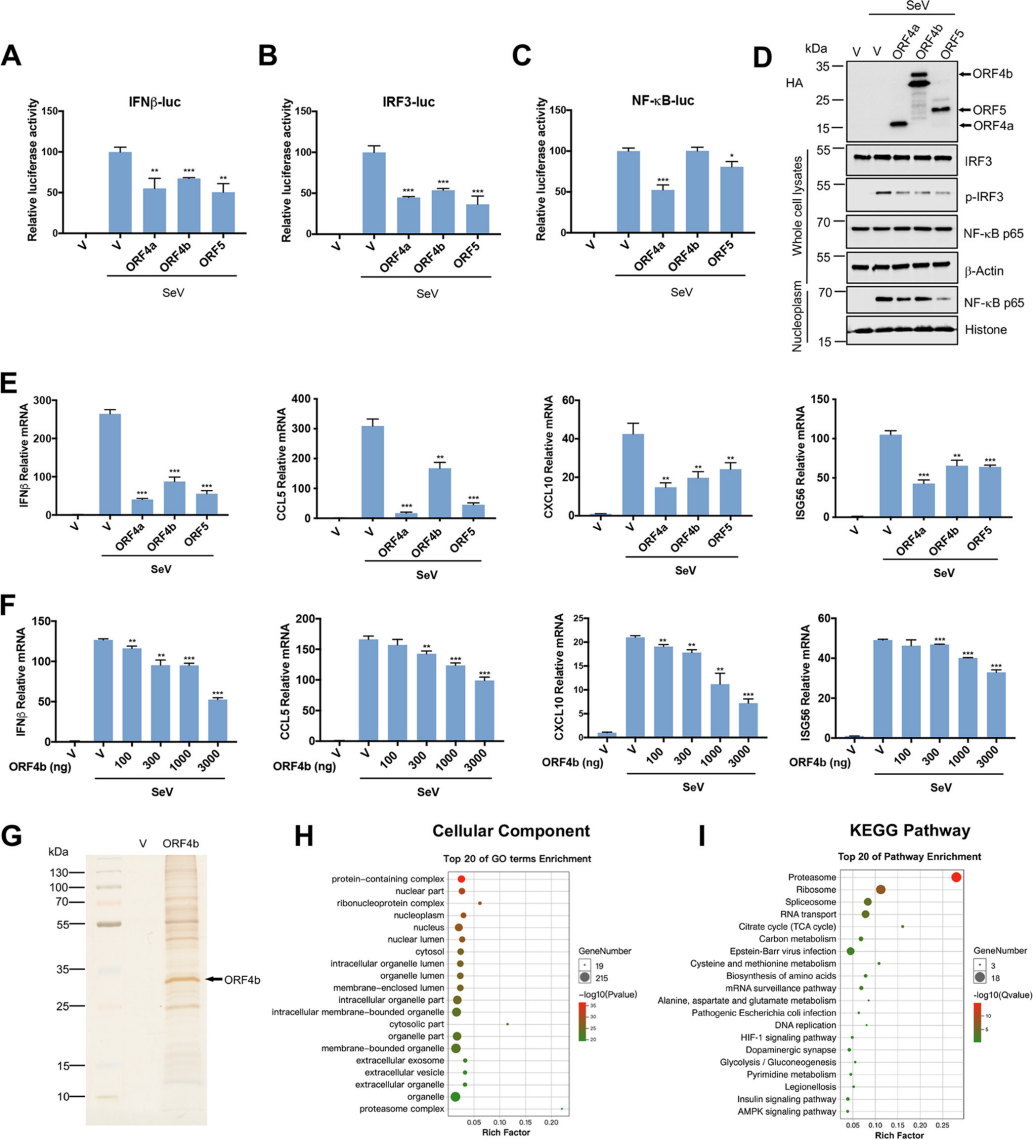
Identifications of MERS-CoV ORF4b functions and its host’s interacting proteins. (A to C) HEK293T cells were cotransfected with expression plasmids for MERS-CoV accessory proteins as indicated and pRL-TK plasmids, together with plasmids expressing firefly luciferase under the control of the IFN-β promoter (A), IRF3 responsive element (B), or NF-κB responsive element (C). Twenty-four hours after transfection, the cells were infected with Sendai virus (SeV) (100 HAU/mL) for 12 h and lysed for the dual-luciferase assay. (D) HEK293T cells were transfected with plasmids expressing HA-tagged MERS-CoV accessory proteins. Twenty-four hours after transfection, the cells were infected with SeV for 12 h and collected for Western blotting. The levels of the indicated proteins were detected with relevant antibodies. (E) HEK293T cells were transfected with empty vector or plasmids expressing the indicated viral proteins. At 24 h posttransfection, cells were infected with SeV (100 HAU/mL) for 12 h. Total RNA was extracted, reverse transcribed, and analyzed by real-time PCR with primers specific for IFN-β, CCL5, CXCL10, and ISG56. (F) HEK293T cells were transfected with an empty vector or increasing amounts of plasmids expressing ORF4b. Twenty-four hours later, cells were infected with SeV for 12 h. The cells were used to extract the total RNA for real-time PCR. (G) HEK293T cells were transfected with empty vector or Flag-ORF4b-expressing plasmid and collected at 48 h posttransfection. Cells were lysed, and whole-cell lysates were immunoprecipitated by anti-Flag beads. Proteins were eluted and detected by silver staining. (H) Cellular Component enrichment analysis of the interacting proteins of ORF4b; (I) KEGG enrichment analysis of the interacting proteins of ORF4b. Error bars indicate SD from technical triplicates. Statistical significance was calculated using an unpaired, two-tailed Student’s *t* test. *, *P* < 0.05; **, *P* < 0.01; ***, *P* < 0.001.

Genome comparison of SARS-CoV, MERS-CoV, and SARS-CoV-2 revealed that MERS-CoV had fewer accessory proteins than SARS-CoV and SARS-CoV-2, albeit it had the highest lethality rate. Among them, three accessory proteins, ORF4a, ORF4b, and ORF5, are specific to MERS-CoV, which may be associated with its high lethal activity (40, 41). To verify the pathological roles of the unique accessory proteins of MERS-CoV, the degree of interferon induction and suppression was assessed by quantifying the promoter activity of the human *IFN*β gene. All three accessory proteins demonstrated resistance to the promoter activity of IFN*-*β (Fig. 1A). More importantly, we aimed to investigate how these viral proteins disrupt the host antiviral signaling pathway. Since IFN regulatory factor 3 (IRF3) and NF-κB are two nuclear transcription factors necessary for interferon production, we examined the effects of the accessory proteins on the activation of IRF3 and NF-κB with Sendai Virus (SeV) stimulation, a common innate immune pathway activation method. ORF4a and ORF5 prevented the activation of these two factors, while ORF4b could only inhibit IRF3-mediated induction of IFN-β (Fig. 1B and C). Meanwhile, we found that ORF4a, ORF4b, and ORF5 could inhibit the phosphorylation of IRF3 when SeV activated the innate immune pathway. However, only ORF4a and ORF5 could attenuate the entry of phosphorylated NF-κB into the nucleus (Fig. 1D). Further experiments revealed that all three accessory proteins could inhibit the mRNA production of well-known downstream cytokines of IRF3 signaling, including IFN-β, CCL5, CXCL10, and a member of the interferon-stimulated gene family, ISG56, in response to SeV (Fig. 1E), poly(I:C) (see Fig. S1A in the supplemental material), and N-terminal RIG-I (Fig. S1B). We then examined the relationship between ORF4b protein level and its anti-immunity ability. The mRNA levels of several antiviral cytokines decreased with increasing amounts of ORF4b protein level, suggesting that ORF4b counteracted host immune responses in a dose-dependent manner (Fig. 1F).

ORF4b protein contained a nuclear localization signal (NLS) that mediated its entry into the nucleus, and inhibited interferon production in both the cytoplasm and nucleus (16, 41). Since ORF4b seemed to have noteworthy participation in the pathogenicity of MERS-CoV, we wanted to further explore and evaluate its other properties. We pulled down ORF4b protein from HEK293T cells, and the silver staining showed multiple possible ORF4b-interacting proteins in host cells (Fig. 1G). Then the immunoprecipitated samples were analyzed by mass spectrometry (MS) to identify possible ORF4b-interacting proteins, and each group analysis was repeated twice (Table S1). Moreover, we performed Gene Ontology (GO) enrichment analysis (Fig. 1H; Fig. S1C and D) and Kyoto Encyclopedia of Genes and Genomes (KEGG) enrichment analysis (Fig. 1I) of ORF4b-interacting proteins. Also, a KEGG enrichment network diagram was constructed to get a global view of the relationships between the pathways (Fig. S1E). The results showed that the interacting proteins of ORF4b were significantly enriched in the proteasome pathway, which implied that ORF4b protein was likely an unstable protein that could be degraded by the proteasome in the host.

### ORF4b is degraded by the ubiquitin-proteasome system.

**FIG 2:**
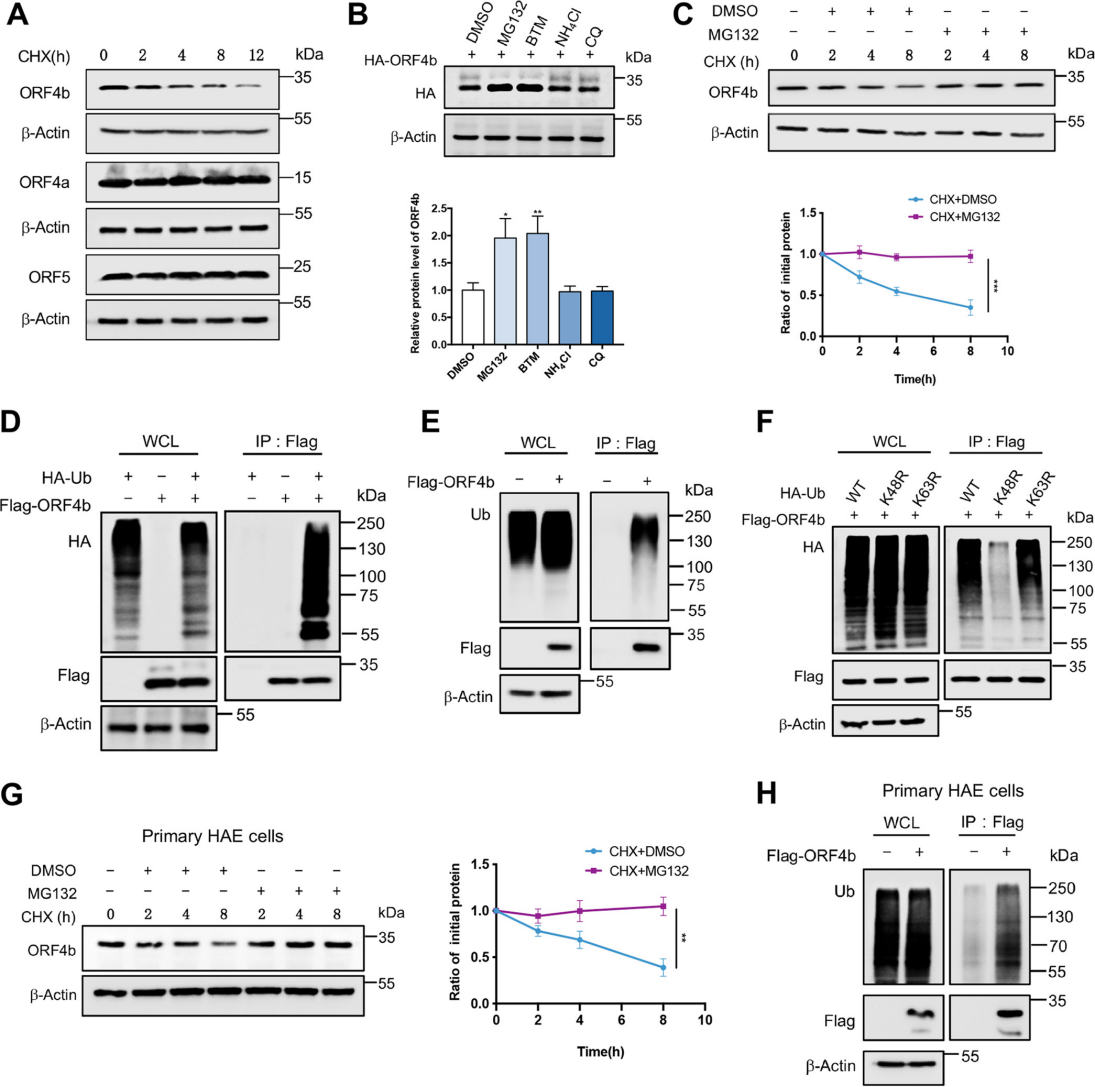
ORF4b is degraded by the ubiquitin-proteasome system. (A) HEK293T cells in a 6-cm dish were transfected with the indicated HA-tagged plasmids. Twelve hours later, the cells were split evenly into the wells of 12-well plates and treated with CHX (30 μg/mL) when they reached 100% confluence. Cells were collected at the indicated times to detect the level of viral protein by anti-HA antibody. (B) HEK293T cells transfected with the indicated plasmids were treated with dimethyl sulfoxide (DMSO), MG132 (20 μM), bortezomib (BTM) (10 μM), chloroquine (CQ) (20 μM), and NH_4_Cl (10 mM) for 8 h before collection. The protein level of ORF4b was detected by Western blotting (top). Quantification of ORF4b protein levels relative to β-actin is shown. Results are shown as mean ± SD (*n* = 3 independent experiments). *, *P < *0.05, and **, *P < *0.01, by Student's *t* test (bottom). (C) HEK293T cells transfected with HA-ORF4b expressing plasmid were cotreated with CHX (30 μg/mL) and DMSO or cotreated with CHX (30 μg/mL) and MG132 (20 μM). Cells were collected at the indicated times for Western blotting by anti-HA antibody (top). Quantification of ORF4b protein levels relative to β-actin is shown. Results are shown as mean ± SD (*n* = 3 independent experiments). ***, *P < *0.001 by two-way analysis of variance (ANOVA) (bottom). (D and E) HEK293T cells cotransfected with HA-ubiquitin and Flag-ORF4b (D) or transfected with Flag-ORF4b alone (E) were treated with MG132 for 8 h before collection. The whole-cell lysates were incubated with anti-Flag beads and used for Western blotting with anti-HA or anti-ubiquitin antibodies to detect the polyubiquitination chain of ORF4b. (F) The ORF4b ubiquitination linkage was analyzed in HEK293T cells transfected with ORF4b and the indicated ubiquitin-WT, Lys-48→Arg, and Lys-63→Arg plasmids. The whole-cell lysates were subjected to pulldown with anti-Flag beads and Western blotting to detect the polyubiquitination chain of ORF4b. (G) The primary human airway epithelial (HAE) cells were transfected with Flag-ORF4b-expressing plasmids and treated and collected as indicated for Western blotting with anti-Flag antibody (left). Quantification of ORF4b protein levels relative to β-actin is shown as mean ± SD (*n* = 3 independent experiments). **, *P < *0.01 by two-way ANOVA (right). (H) The primary HAE cells were transfected with Flag-ORF4b-expressing plasmids and lysed to analyze the polyubiquitination chain of ORF4b.

Based on bioinformatics analysis, we treated HEK293T cells overexpressing viral proteins with cycloheximide (CHX) to inhibit protein synthesis and determine the protein half-life. ORF4b protein turned out to be degraded intracellularly over time, while the other two proteins, ORF4a and ORF5, remained stable without degradation, which implied that ORF4b was an unstable protein in cells (Fig. 2A). Intracellular degradation of exogenous proteins is mainly carried out by two pathways, including the ubiquitin proteasome pathway and the lysosomal pathway (42). To verify if ORF4b was exclusively degraded by the proteasome, we treated cells overexpressing ORF4b protein with the proteasome inhibitors MG132 and bortezomib (BTM) and the lysosomal inhibitors ammonium chloride (NH_4_Cl) and chloroquine (CQ). Proteasome inhibition significantly increased the protein levels of ORF4b, whereas lysosomal inhibitors had no effect (Fig. 2B). The protein half-life assay further confirmed that inhibitors of proteasome-mediated proteolysis—but not the lysosomal inhibitors—could stabilize ORF4b (Fig. 2C; Fig. S2A to C). Because MERS-CoV caused respiratory diseases and gastrointestinal disorders, the stability of ORF4b protein was also explored in some lung-associated and intestine-associated cells, including those of the Calu3, A549, BEAS-2B, HCT116, HT29, and NCM460 cell lines. Similar to HEK293T cells, ORF4b was also degraded by the proteasome in these cell lines (Fig. S2D). All of these results indicated that ORF4b could be degraded in various cell lines exclusively through the proteasome pathway with no apparent involvement of lysosome-mediated proteolysis.

If a protein is recognized and degraded by the proteasome, it should be ubiquitinated first. We found that both overexpressed and endogenous ubiquitin could link with ORF4b protein to form polyubiquitin chains (Fig. 2D and E), which further manifested that ORF4b underwent ubiquitination in host cells. Generally, the fate of a substrate protein following its ubiquitination depends mainly on the type of the conjugation of specific polyubiquitin chains. To find out the type of ubiquitination of ORF4b, we coexpressed Flag-ORF4b with hemagglutinin (HA)-ubiquitin wild type or lysine-specific mutants. The Lys-48→Arg mutant of ubiquitin lost the ability to link to ORF4b protein, but not the Lys-63→Arg mutant (Fig. 2F). We also verified ubiquitinated degradation of ORF4b protein in primary human airway epithelial (HAE) cells, a more relevant *in vitro* model (Fig. 2G and H). These findings confirmed that K48-linked polyubiquitination mediated the recognition and eventual degradation of ORF4b by the proteasome.

### ORF4b interacts with UBR5.

**FIG 3:**
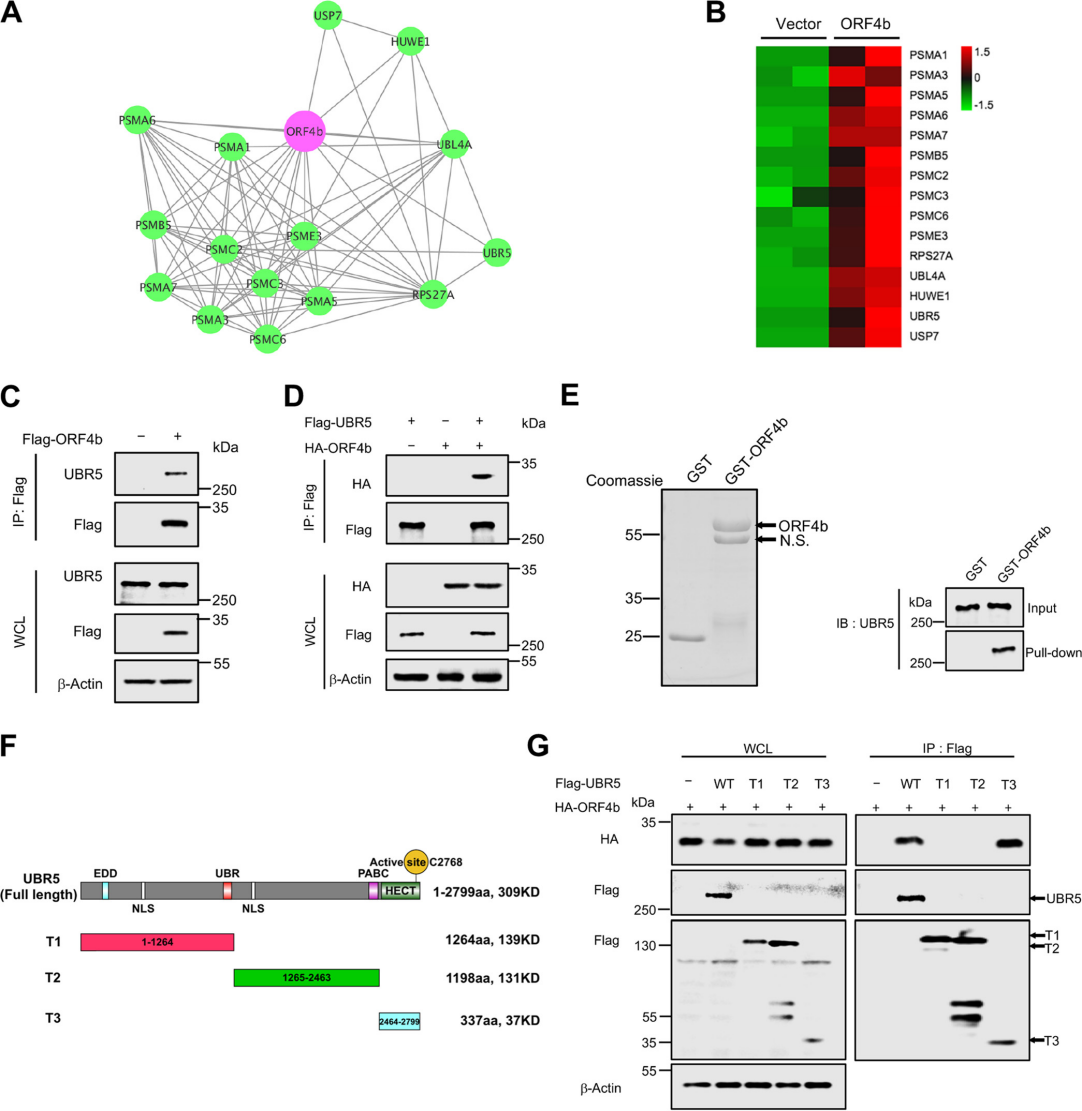
ORF4b interacts with UBR5. (A) The protein-protein interaction network of ORF4b and interacting proteasome subunits and E3 ligase candidates is shown, based on mass spectrometry results. (B) A heat map shows the ORF4b-interacting proteins’ enrichment in the proteasome pathway and E3 ligase family. (C) HEK293T cells transfected with empty vector or plasmid containing Flag-ORF4b were collected at 48 h after transfection. Cells were lysed and immunoprecipitated with anti-Flag beads. Proteins in the whole-cell lysates and immunoprecipitate were blotted with the indicated antibodies. (D) Flag-UBR5- and HA-ORF4b-expressing plasmids were cotransfected into HEK293T cells. Forty-eight hours after transfection, cells were lysed and immunoprecipitated with anti-Flag beads. The whole-cell lysates and immunoprecipitated proteins were detected with anti-HA and anti-Flag antibodies by Western blotting. (E) GST and GST-ORF4b were purified from *E. coli* and analyzed by Coomassie staining (left). The beads binding the target proteins were incubated with the whole-cell lysates from HEK293T cells, and the pulldown proteins were detected with anti-UBR5 antibody by immunoblotting (right). N.S., nonspecific. (F) The structural domains of UBR5 are diagrammed. (G) HEK293T cells were transfected with the indicated plasmids. Whole-cell lysates were precipitated with anti-Flag beads. Whole-cell lysates and precipitated proteins were analyzed by Western blotting with the indicated antibodies.

Protein ubiquitination requires the sequential action of E1, E2, and E3 enzymes to transfer ubiquitin to the target protein. Of these, the E3 ligases play a major role in recognizing specific substrates. To this end, we aimed to identify the specific E3 ligase targeting ORF4b. According to the mass spectrometry (MS)-identified interactors of ORF4b, we constructed a protein-protein network based on the proteasome subunits and E3 ligases (Fig. 3A). A heat map based on the number of peptides identified for each protein showed two high-confidence candidates, UBR5 and HUWE1 (Fig. 3B; Fig. S3A and B). In further experimental validation, we found that ORF4b protein was coprecipitated with endogenous and exogenous UBR5 (Fig. 3C and D; and Fig. S3C), but not with HUWE1 (Fig. S3D and E). Similarly, an *in vitro* glutathione *S*-transferase (GST) pulldown assay further corroborated the interaction between UBR5 and ORF4b (Fig. 3E). UBR5 is a multidomain protein. To further map the interaction of ORF4b with the domains of UBR5, we constructed three truncated UBR5 variants, termed T1 (amino acids [aa] 1 to 1264), T2 (aa 1265 to 2463), and T3 (aa 2464 to 2799) (Fig. 3F). The truncated polypeptides T1 and T2 failed to bind with ORF4b, and only T3 containing the HECT domain was sufficient for the interaction (Fig. 3G). Taken together, ORF4b interacted with UBR5 through its HECT domain.

### UBR5 mediates the ubiquitination and degradation of ORF4b.

**FIG 4:**
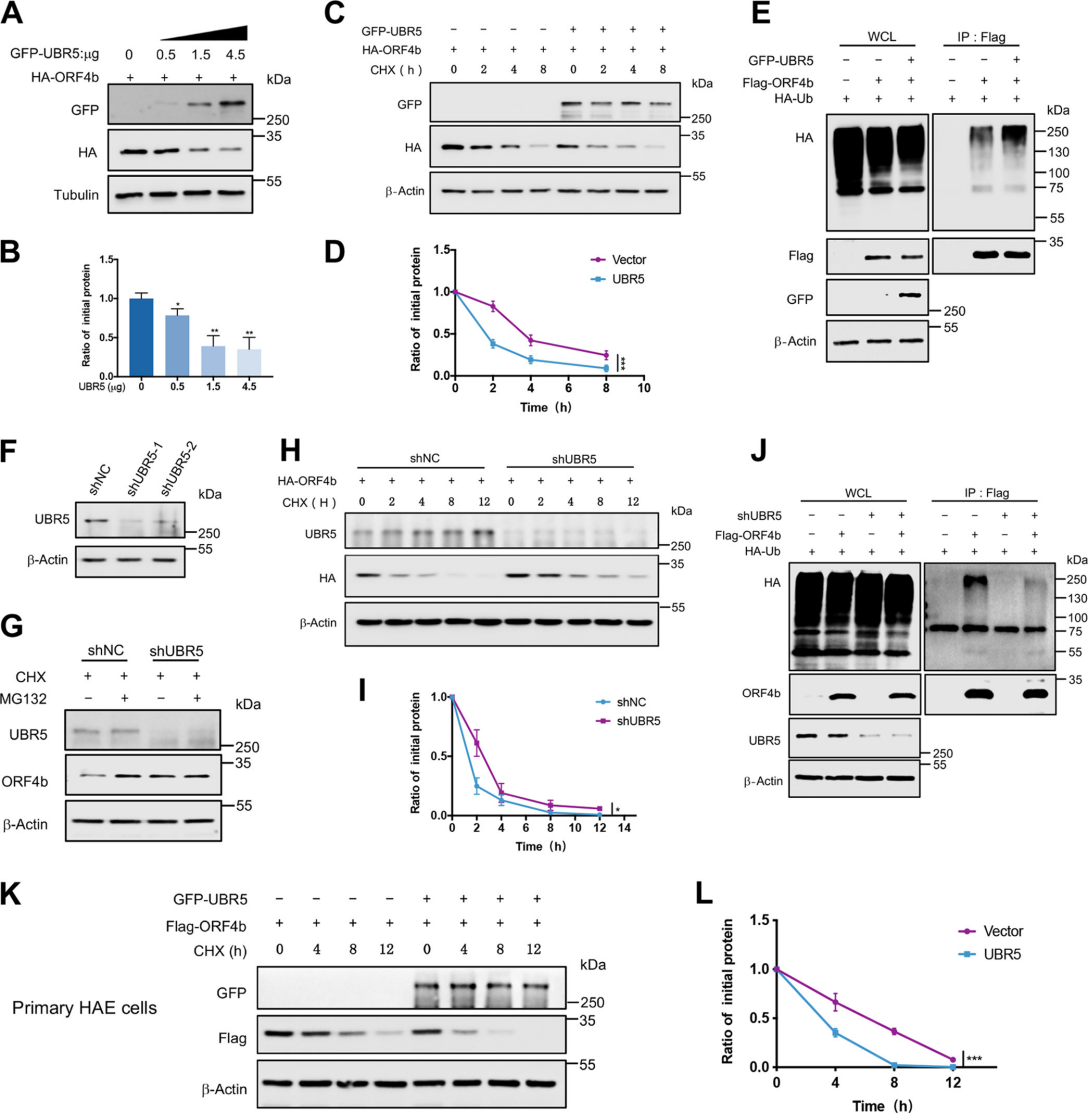
UBR5 mediates the ubiquitination and degradation of ORF4b. (A and B) HEK293T cells transfected with ORF4b-expressing plasmid were split into the wells of a 12-well plate and were then transfected with increasing amounts of plasmids containing UBR5. The cells were collected at 48 h posttransfection to analyze the protein level of ORF4b by Western blotting (A). Quantification of ORF4b protein levels relative to β-actin is shown. Results are shown as mean ± SD (*n* = 3 independent experiments). *, *P < *0.05, and **, *P < *0.01, by Student's *t* test (B). (C and D) The plasmids containing HA-ORF4b, together with empty vector or UBR5-expressing plasmids were transfected into HEK293T cells and were split into the wells of a 12-well plate. Twenty-four hours later, the cells were treated with CHX (30 μg/mL) and collected at the indicated times to detect the protein level of ORF4b (C). Quantification of ORF4b protein levels relative to β-actin is shown. Results are shown as mean ± SD (*n* = 3 independent experiments). ***, *P < *0.001 by two-way ANOVA (D). (E) ORF4b ubiquitination was analyzed in HEK293T cells transfected with ORF4b together with UBR5 or not. (F) Immunoblots of UBR5 from HEK293T cells infected with lentivirus containing control or shRNA targeting UBR5 for 72 h. (G) HEK293T cells stably expressing shNC and shUBR5 were transfected with plasmid containing ORF4b and were treated with MG132 (20 μM) for 8 h. Cells were collected to detect the protein level of ORF4b by Western blotting. (H and I) HEK293T cells stably expressing UBR5 shRNA and shNC were transfected with ORF4b-expressing plasmid and split evenly into the wells of a 12-well plate. Cells were treated with CHX (30 μg/mL) and collected at the indicated times to test the protein level of ORF4b (H). Quantification of ORF4b protein levels relative to β-actin is shown. Results are shown as mean ± SD (*n* = 3 independent experiments). *, *P < *0.05 by two-way ANOVA (I). (J) ORF4b ubiquitination was analyzed in UBR5 shRNA and shNC-transduced HEK293T cells, transfected with HA-ubiquitin together with Flag-ORF4b or not. (K and L) The plasmids containing Flag-ORF4b together with empty vector or UBR5-expressing plasmids were transfected into the primary HAE cells. The cells were treated and collected as indicated to test the half-lives of ORF4b (K). Quantification of ORF4b protein levels relative to β-actin is shown as mean ± SD (*n* = 3 independent experiments). ***, *P < *0.001 by two-way ANOVA (L).

After verifying the association of UBR5 with ORF4b, we sought to test whether UBR5 could perform as a E3 ligase and regulate the degradation of ORF4b. First, we overexpressed an increasing amount of UBR5 in HEK293T cells. As UBR5 expression increased, the protein level of ORF4b decreased (Fig. 4A and B). Meanwhile, the half-life of ORF4b protein decreased and the rate of degradation increased when cells were treated with CHX in the presence of overexpressed UBR5 (Fig. 4C and D). Next, we went on to test whether UBR5 could ubiquitinate ORF4b and found that ectopic expression of UBR5 increased the polyubiquitination of ORF4b (Fig. 4E). Moreover, we selected two short hairpin RNAs (shRNAs) targeting UBR5 and constructed cell lines stably expressing these shRNAs (Fig. 4F). After the knockdown of UBR5, MG132 treatment no longer increased the protein level of ORF4b (Fig. 4G). In addition, the stability assay with CHX treatment further confirmed that the half-life of ORF4b increased (Fig. 4H and I) and fewer polyubiquitin chains of ORF4b were formed (Fig. 4J) when UBR5 was knocked down. In primary HAE cells, overexpression of UBR5 also significantly enhanced the degradation of ORF4b protein (Fig. 4K and L). Previously, it was reported that the C2768, located on the HECT domain, was a single residue essential for the catalytic activity of UBR5 (Fig. S4A) (43). With the increase in expression of UBR5-C2768A (a mutant bearing a cysteine-to-alanine substitution in its catalytic site), the protein level of ORF4b was not reduced (Fig. S4B). Compared with overexpression of UBR5-WT, overexpression of enzymatic activity-deficient UBR5-C2768A did not attenuate the protein level of ORF4b (Fig. S4C and D). Furthermore, we performed an *in vitro* ubiquitination assay using GST-ORF4b purified from bacteria, Flag-UBR5-WT, and Flag-UBR5-C2768A purified from HEK293T cells (Fig. S4E). These results indicated that UBR5 mediated ORF4b ubiquitination to direct its degradation by the proteasome.

### UBR5 induces the degradation of ORF4b in both cytoplasm and nucleoplasm.

**FIG 5:**
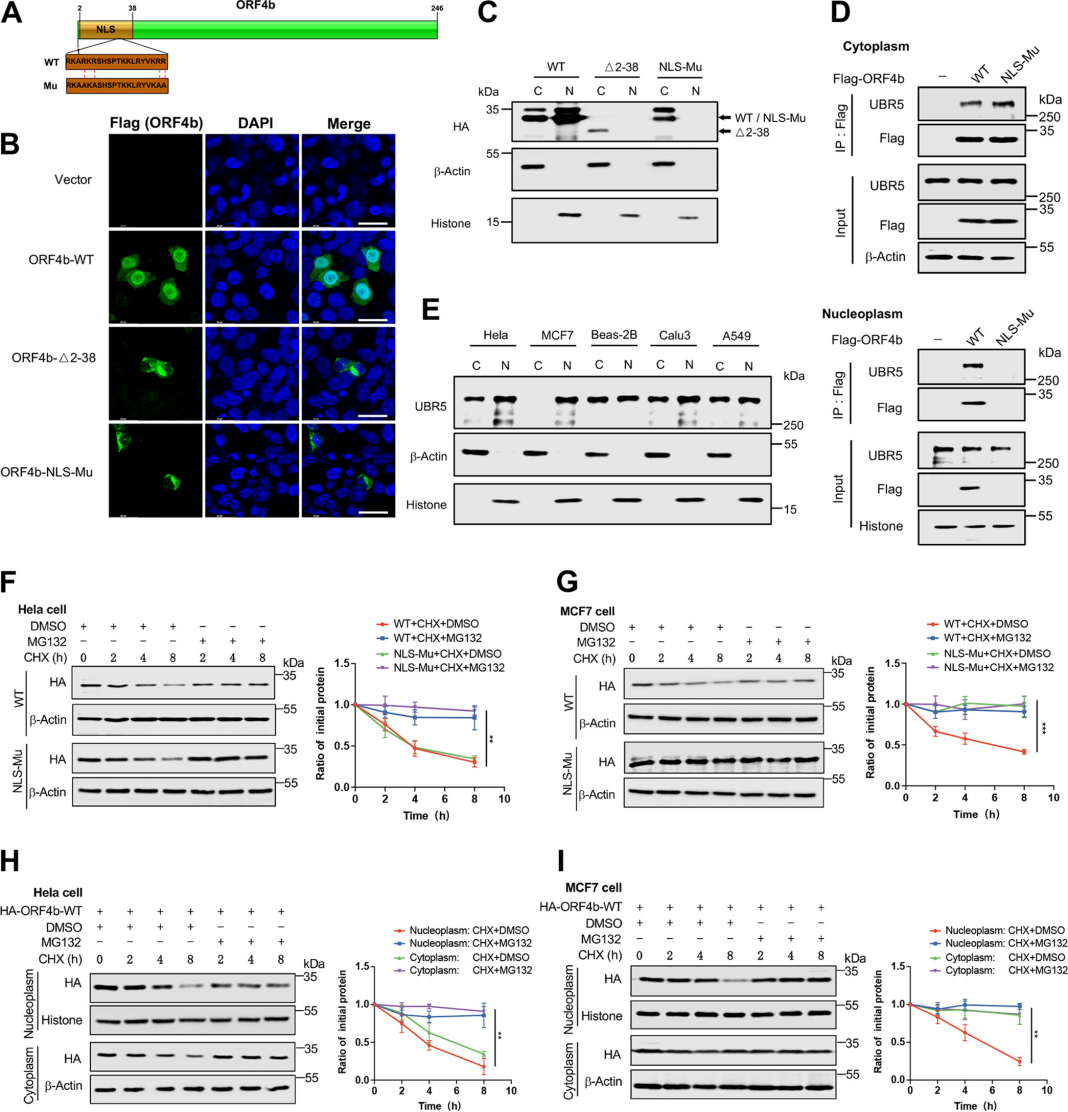
UBR5 induces the degradation of ORF4b in both cytoplasm and nucleoplasm. (A) The pattern diagram shows the mutations of arginine to alanine in the basic amino acid group of ORF4b nuclear localization signal. (B) HeLa cells transfected with the indicated plasmids were analyzed by confocal microscopy. The Flag-tagged ORF4b WT and mutants were labeled with anti-Flag antibody (green). Cell nuclei were stained using DAPI (blue). Representative images are shown. Scale bars, 25 μm. (C) HEK293T cells transfected with HA-tagged ORF4b and mutants were used for nuclear and cytoplasmic protein extraction. The indicated proteins in cytoplasm and nucleoplasm were detected by Western blotting. (D) The proteins of cytoplasm and nucleoplasm were extracted from HEK293T cells transfected with the indicated plasmids. The cytoplasmic and nuclear proteins were subjected to pulldown with anti-Flag beads. ORF4b and endogenous UBR5 were detected with the indicated antibodies by Western blotting. (E) The cytoplasmic and nuclear proteins of HeLa, MCF7, BEAS-2B, Calu3, and A549 cells were extracted. The protein levels of UBR5 were detected by Western blotting. (F and G) HeLa (F) and MCF7 (G) cells transfected with indicated plasmids expressing HA-ORF4b-WT and HA-ORF4b-NLS-Mu (nuclear location signal mutant) were cotreated with CHX (30 μg/mL) and DMSO or CHX (30 μg/mL) and MG132 (20 μM). Cells were collected to detect the indicated protein level by Western blotting. (H and I) HeLa (H) and MCF7 (I) cells transfected with HA-tagged ORF4b were cotreated with CHX and DMSO or CHX and MG132. Cells were collected and used for nuclear and cytoplasmic protein extraction. The levels of the indicated proteins in both cytoplasm and nucleoplasm were analyzed by Western blotting. Error bars indicate SD from technical triplicates. Quantification of ORF4b protein levels relative to β-actin is shown. **, *P* < 0.01, and ***, *P* < 0.001, by two-way ANOVA.

In contrast to other accessory proteins of MERS-CoV, ORF4b was unique because it could enter the nucleus and inhibit interferon production in both cytoplasm and nucleoplasm (16). ORF4b could localize in the nucleus through the NLS enriched with positively charged arginine (R) and lysine (K). The RKR at positions 22 to 24 and the KRR at positions 36 to 38 played a significant role in the nuclear localization of ORF4b (44). Previously, removing the amino acids 2 to 38 containing the NLS prevented ORF4b from entering the nucleus (16). However, the NLS contained multiple lysine residues that were potential ubiquitination sites. Aiming to understand the mechanism of ORF4b ubiquitination in both cytoplasm and nucleoplasm, a mutant ORF4b with mutations in NLS (NLS-Mu) that replaced two sets of positively charged arginines with alanines was constructed in our study, attenuating the overall charge of the NLS (Fig. 5A). We also constructed the mutant ORF4b-Δ2-38, which lacked the whole NLS, by deleting N-terminal aa 2 to 38. With a similar effect to ORF4b-Δ2-38, NLS-Mu also localized only to the cytoplasm, while ORF4b-WT localized to both cytoplasm and nucleus (Fig. 5B and C).

UBR5 could also localize to both cytoplasm and nucleus with the presence of two NLSs (45, 46). Hence, verification of whether ORF4b could interact with UBR5 in both cytoplasm and nucleus was needed. The results showed that ORF4b-WT could interact with UBR5 in both locations, while the NLS-Mu could only interact with UBR5 in the cytoplasm (Fig. 5D). In most cells, UBR5 was present in both cytoplasm and nucleoplasm, except in MCF7 cells, in which UBR5 localized only in the nucleus (Fig. 5E). Furthermore, we tested whether UBR5-mediated degradation of ORF4b occurred in both cytoplasm and nucleoplasm. In HeLa cells where UBR5 was localized in the cytoplasm and nucleus, both ORF4b-WT and NLS-Mu could be degraded significantly (Fig. 5F). On the contrary, degradation of ORF4b-WT, but not NLS-Mu, was observed in MCF7 cells, whose UBR5 is exclusively localized in the nucleus (Fig. 5G). Consistently, immunoblotting of proteins extracted from nucleoplasm and cytoplasm revealed that in HeLa cells, ORF4b could be degraded in both regions (Fig. 5H). In MCF7 cells, only ORF4b in the nucleus could be degraded, while the protein level of ORF4b in the cytoplasm remained stable (Fig. 5I). All in all, these results indicated that UBR5 was the unique E3 ligase that induced the ubiquitination and degradation of ORF4b protein in both cytoplasm and nucleoplasm.

### Ubiquitination-resistant mutation restores the stability of ORF4b.

**FIG 6:**
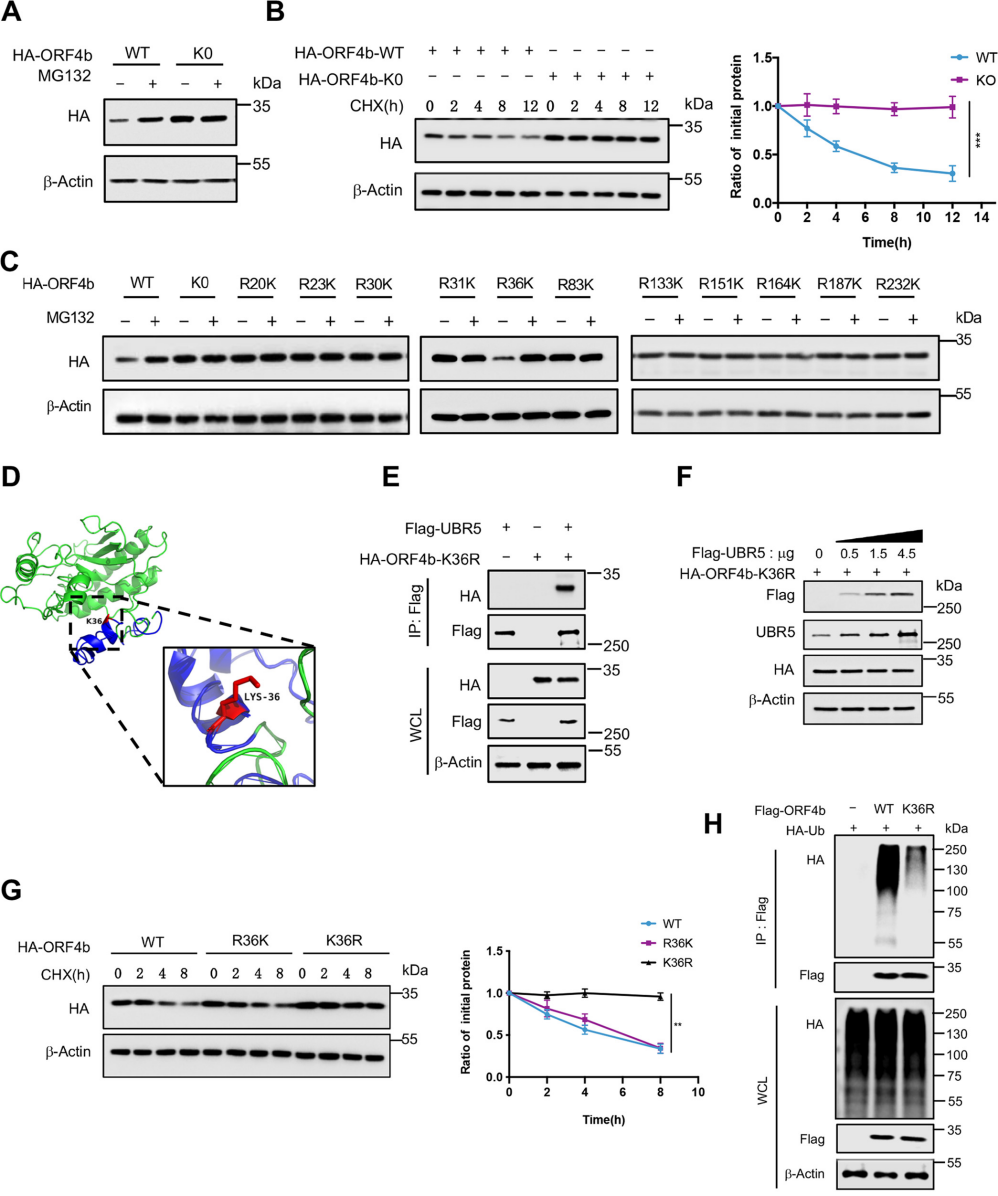
Ubiquitination-resistant mutation restores the stability of ORF4b. (A) The plasmid expressing ORF4b-WT or ORF4b-K0 was transfected into HEK293T cells. The cells were treated with MG132 (20 μM) or not for 8 h before collection. The protein level of ORF4b was detected with anti-HA antibody by Western blotting. (B) HEK293T cells transfected with plasmids containing ORF4b-WT and ORF4b-K0 (in which all lysine was mutated to arginine) were cotreated with CHX (30 μg/mL) and DMSO or CHX (30 μg/mL) and MG132 (20 μM). Cells were collected to detect the level of indicated protein (left). Quantification of the ORF4b protein level was normalized to β-actin. Results are shown as mean ± SD (*n* = 3 independent experiments). ***, *P < *0.001 by two-way ANOVA (right). (C) HEK293T cells were transfected with ORF4b-WT, ORF4b-K0, and the ORF4b single mutant with arginine changed to lysine based on the K0 mutant. Cells with different transfection were split evenly and treated with MG132 (20 μM) or not for 8 h before collection. The indicated protein levels were detected by Western blotting. (D) A PDB file of ORF4b was modeled by the I-TASSER server from the Zhang laboratory. Visualization of the three-dimensional model of ORF4b was performed with PyMOL. K36 is located on the surface (red) and in the NLS (blue). (E) The plasmids expressing Flag-UBR5 and HA-ORF4b-K36R were cotransfected into HEK293T cells. The whole-cell lysates were immunoprecipitated with anti-Flag beads. The proteins were detected with the indicated antibodies by Western blotting. (F) HEK293T cells transfected with the ORF4b-K36R-expressing plasmid were split into the wells of a 12-well plate and then were transfected with increasing amounts of plasmids containing UBR5. The cells were collected at 48 h posttransfection to analyze the protein level of ORF4b by Western blotting. (G) Half-life analysis of ORF4b-WT, ORF4b-R36K, and ORF4b-K36R. HEK293T cells transfected with indicated plasmid were split evenly into the wells of a 12-well plate. Cells were treated with CHX (30 μg/mL) and collected at the indicated times to detect the level of target proteins (left). Quantification of the ORF4b protein level was normalized to β-actin. Results are shown as mean ± SD (*n* = 3 independent experiments). **, *P < *0.01 by two-way ANOVA (right). (H) Ubiquitination analysis of ORF4b-WT and ORF4b-K36R was performed with HEK293T cells transfected with the indicated plasmids. The polyubiquitination chains were detected by anti-HA antibody.

So far, we have demonstrated the ubiquitin-mediated regulation of ORF4b by the E3 ligase UBR5. Next, we sought to further identify the ubiquitination site on ORF4b. Since ubiquitination frequently occurs on lysine residues of the target protein, we first mutated all 11 lysine residues of ORF4b to arginine, termed K0. In contrast to the WT, MG132 failed to increase the protein level of K0 (Fig. 6A). When the cells overexpressing ORF4b-WT and ORF4b-K0 were treated with CHX, we found ORF4b-K0 remained stable over time compared with ORF4b-WT (Fig. 6B). Next, we reverse mutated R back to K based on K0 to determine the specific ubiquitination site of ORF4b. The results showed that the mutation R36K was sufficient to decrease the ORF4b protein level and that proteasome inhibition by MG132 could stabilize such mutant (Fig. 6C). This implied that K36 was likely to be the ubiquitination site of ORF4b. We then modeled the ORF4b protein structure and found the K36 was located on the surface and embedded within the nuclear localization signal (Fig. 6D). Although the K36R mutation did not abrogate the interaction between UBR5 and ORF4b, the gradient overexpression of UBR5 did not affect the protein level of this mutant (Fig. 6E and F). The half-life analysis showed that the protein levels of the WT and R36K mutant decreased over time compared with those of other R-K mutants, whereas K36R was not degraded (Fig. 6G; Fig. S5A). *In vivo* ubiquitination analysis also showed that K36R formed significantly fewer polyubiquitin chains than the WT (Fig. 6H). Furthermore, genomic analysis of MERS-CoV strains from different countries showed that the ORF4b protein was highly conserved at K36 (Fig. S5B). The above results collectively suggested that the ORF4b protein was ubiquitinated at the highly conserved lysine K36.

### UBR5 acts as an anti-MERS-CoV factor in host cells.

**FIG 7:**
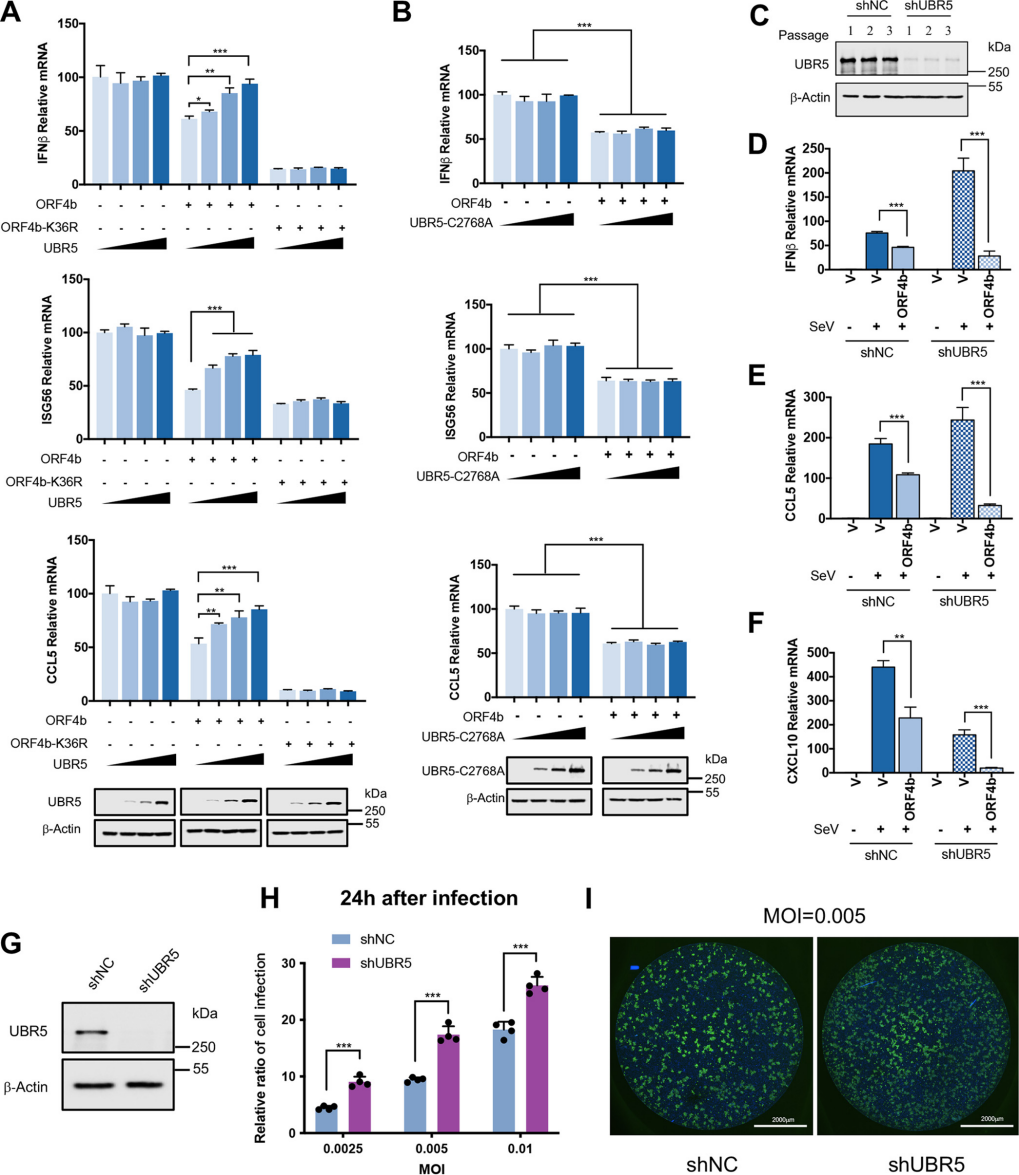
UBR5 acts as an anti-MERS-CoV factor in host cells. (A) Increasing amounts of plasmids containing Flag-UBR5 were transfected into HEK293T cells expressing empty vector, HA-ORF4b-WT, or HA-ORF4b-K36R. Cells were infected with SeV (100 HAU/mL) for 12 h and collected. Thirty percent of the cells were lysed and used for Western blotting (bottom), and the rest were used for RNA extraction and analyzed by real-time PCR with primers specific for the indicated genes. (B) The plasmid containing Flag-UBR5-C2768A was transfected into HEK293T cells expressing empty vector or ORF4b protein in increasing gradients. After being treated with SeV (100 HAU/mL) for 12 h, 30% of the cells were collected to detect the indicated proteins (bottom). Total RNA of the rest cells was extracted, reverse transcribed, and analyzed by real-time PCR with primers specific for target genes. (C) Calu3 cells were infected with lentivirus containing UBR5 shRNA and were selected by puromycin (2 μg/mL) for 72 h. The cell lines were passaged for three generations, and each passage was collected to detect the protein level of endogenous UBR5 by Western blotting. (D to F) The plasmids expressing empty vector or ORF4b were transfected into shNC or shUBR5 Calu3 stable cell lines and infected with SeV (100 HAU/mL) for 12 h. Total RNA was extracted, and the relative mRNA level of IFN-β (D), CCL5 (E), and CXCL10 (F) was analyzed by real-time PCR with specific primers. (G) Huh7 cells were infected with lentivirus containing UBR5 shRNA and were selected by puromycin (2 μg/mL) for 72 h. The cells were collected to detect the protein level of endogenous UBR5 by Western blotting. (H) Huh7 cells were infected with MERS-CoV at the indicated MOIs of 0.0025, 0.005, and 0.01. Twenty-four hours later, an indirect immunofluorescence assay (IFA) was used to detect S protein expression in MERS-CoV-infected cells and the relative ratio of cell infection was analyzed. (I) Huh7 cells infected with MERS-CoV were analyzed by confocal microscopy. Cells were incubated with anti-MERS-CoV S protein antibody, and nuclei were stained with DAPI (blue). Representative images are shown. Scale bars, 2,000 μm. Error bars indicate SD from technical triplicates. Statistical significance was calculated using an unpaired, two-tailed Student’s *t* test: *, *P* < 0.05; **, *P* < 0.01; ***, *P* < 0.001.

So far, we have determined that UBR5 was the specific E3 ligase that induced ubiquitination of ORF4b by forming ubiquitin linkages at K36, leading to its proteasomal degradation. Since the ability of ORF4b to resist host immunity was dose dependent, as shown in the earlier results, we hypothesized that UBR5 might be an antiviral factor in the host to achieve antagonism against MERS-CoV by mediating the degradation of ORF4b. To test this, we overexpressed UBR5 protein by increasing amounts in the HEK293T cells expressing ORF4b-WT or ORF4b-K36R. The results showed that overexpression of UBR5 in the absence of ORF4b had no effect on the production of cellular antiviral cytokines, such as IFN-β, CCL5, and ISG56. While in the cells expressing ORF4b-WT, the inhibitory effect of ORF4b on related cytokines gradually diminished with increasing levels of UBR5 protein. Nevertheless, in the cells expressing ORF4b-K36R, overexpression of UBR5 could not attenuate the inhibitory effect of ORF4b-K36R on cytokines (Fig. 7A). The previous results illustrated that the C2768A mutant, a catalytically dead mutant of UBR5 with no E3 ligase activity, could not mediate the degradation of ORF4b. We then tested whether the catalytic activity of UBR5 was necessary for modulation of ORF4b-mediated suppression of antiviral cytokine production. The gradient overexpression of UBR5-C2768A, like that of UBR5-WT, did not affect cytokine production. More importantly, the C2768A mutation also failed to affect the anti-immunity ability of ORF4b-WT (Fig. 7B). Based on these results, we aimed to further explore the anti-immunity ability of ORF4b in UBR5 knockdown Calu3 stable cell lines (Fig. 7C). In Calu3 shNC cells, ORF4b downregulated the mRNA of IFN-β, CCL5, and CXCL10 by around 2-fold. Strikingly, ORF4b in shUBR5 cells downregulated IFN-β, CCL5, and CXCL10 by around 7- to 9-fold. Knockdown of UBR5, as expected, significantly increased the ability of ORF4b to inactivate host immune defenses (Fig. 7D to F). In more depth, we constructed an Huh7 stable cell line with knockdown of UBR5 (Fig. 7G) and then infected Huh7 cells with MERS-CoV of different multiplicities of infection (MOIs) and analyzed the relative amount of virus by indirect immunofluorescence detection of S protein. Compared with shNC cells, when UBR5 was knocked down, the relative ratio of cell infection was approximately 2-fold (Fig. 7H and I). Taking all of our data together, we propose that the ubiquitin-mediated proteolysis of ORF4b by UBR5 results in the reduction of the intracellular level of ORF4b protein in both cytoplasm and nucleoplasm. This reduction of ORF4b weakens its ability to inhibit the host’s immune responses. Therefore, UBR5 could act as an anti-MERS-CoV host protein to antagonize the immune escape strategy of MERS-CoV.

## DISCUSSION

**FIG 8:**
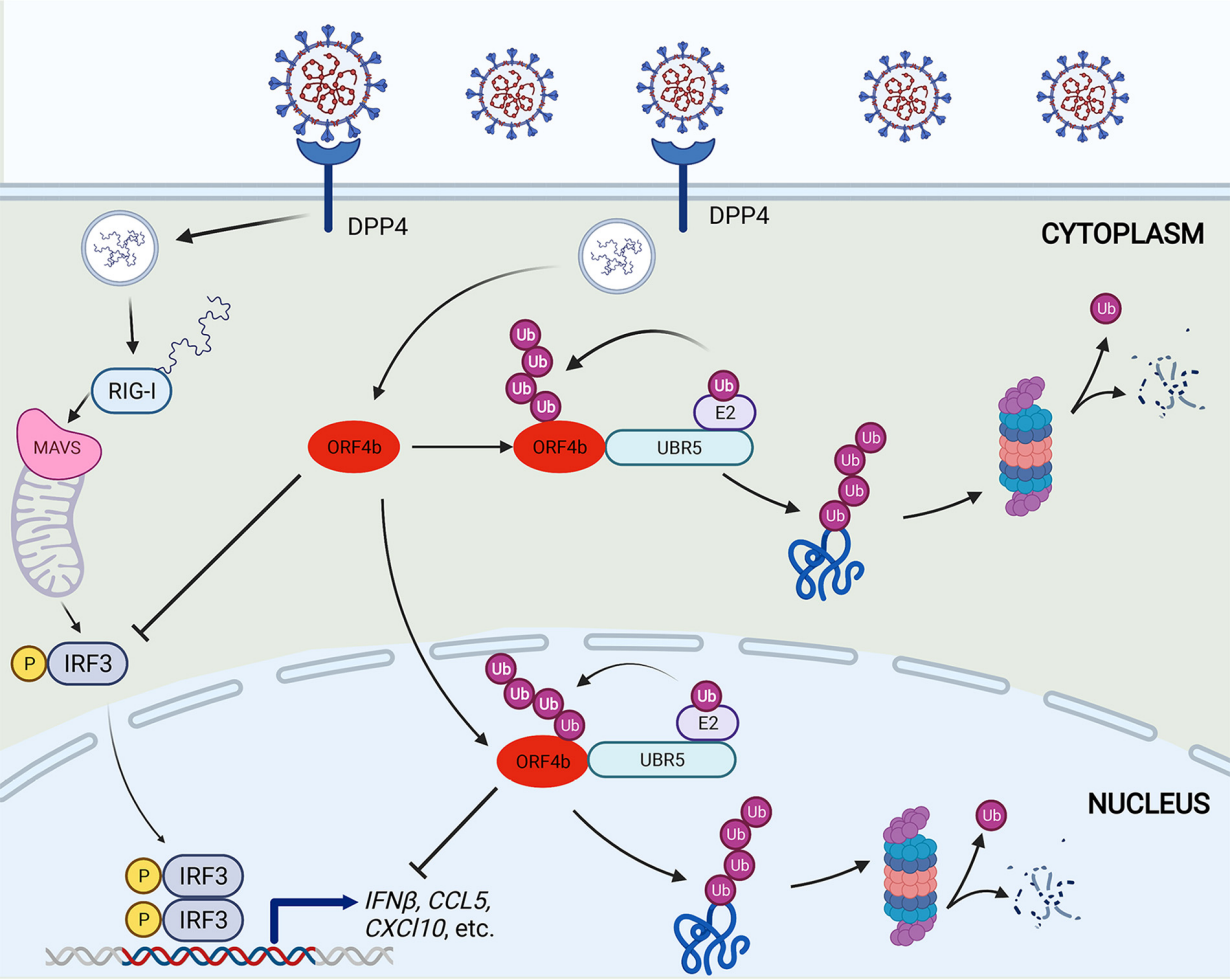
Pattern diagram of UBR5 as an antiviral factor against MERS-CoV by promoting degradation of ORF4b. DPP4, dipeptidyl peptidase 4; RIG-I, retinoic acid-induced gene I; MDA5, melanoma differentiation-associated protein 5; MAVS, mitochondrial antiviral-signaling protein.

ORF4b prevented the activation of IRF3 in response to SeV infection by inhibiting IRF3 phosphorylation and targeted several components of the RIG-I-like receptor (RLR)-mediated IFN signaling pathway, including MDA5, TBK1, and IKKε (14, 16). ORF4b thus serves as the antagonist of the early antiviral interferon responses, but a host strategy to combat this critical anti-immunity ability has not been reported elsewhere. In the present study, we first found that ORF4b was an unstable protein and could be degraded by the ubiquitin proteasome system. Based on our bioinformatic screening, corroborated by various experimental validations, we identified the E3 ligase UBR5 interacted with ORF4b and specifically mediated ORF4b ubiquitination, which in turn allowed it to be degraded by the proteasome. Because UBR5 also possessed a nuclear localization signal similar to that of ORF4b, UBR5 could induce the degradation of ORF4b in both the cytoplasm and nucleus. Additionally, we identified that the lysine 36 on ORF4b, a highly conserved residue in distinct MERS-CoV strains, underwent ubiquitination. Lysine-to-arginine mutation at this site rendered the ORF4b protein more stable with significantly decreased polyubiquitination chains. A deeper study found that MERS-CoV exhibited greater replication ability when UBR5 was knocked down in Huh7 cells. Taken together, UBR5 could attenuate the anti-immunity ability of ORF4b by inducing its ubiquitination and degradation (Fig. 8). These findings demonstrated that UBR5 was an intracellular anti-MERS-CoV protein that can weaken the immune escape of MERS-CoV and exerted an antagonistic effect on MERS-CoV pathogenesis.

Ubiquitination requires a series of reactions of ubiquitin activating enzyme E1, ubiquitin binding enzyme E2, and ubiquitin ligase E3 to transfer ubiquitin to the target protein (47). Among hundreds of E3 ligases, UBR5 was known as a member of the N-end rule ubiquitination pathway and belonged to the HECT family. It was aberrantly expressed in a variety of tumors, regulating tumor biology in terms of the cell cycle, apoptosis, invasion, and metastasis (46, 48–50). In viral infections, UBR5 could target and degrade human T cell leukemia virus type 1 (HTLV-1) antisense-derived protein (HBZ) to maintain the proliferative phenotype of transformed T-cell lines (51). UBR5 could also be utilized by HPV E6 to destabilize tumor suppressor TIP6o and then affect DNA damage response and cell apoptosis (52). However, the relationship between UBR5 and coronaviruses has not been reported elsewhere. Our study has exemplified for the first time that UBR5 could act as an antagonistic factor against MERS-CoV and diminish the replicative capacity of MERS-CoV by specifically inducing the degradation of ORF4b protein.

During viral infection, the virus and the host are in a mutually antagonistic state (53), where protein posttranslational modification, especially ubiquitination, plays an influential role (17, 20). The ubiquitination of key factors in the innate immune pathway during viral infection has been abundantly studied (25, 26, 54). However, the ubiquitination of coronaviral proteins remains largely unexplored. Among other coronaviruses, ORF8b and NSP16 of SARS-CoV and ORF9c of SARS-CoV-2 have been reported to be ubiquitinated and degraded by the ubiquitin proteasome system (36–38). However, the viral proteins of the highly pathogenic virus MERS-CoV have not been thoroughly investigated until now, despite its considerable pandemic risks.

Given ubiquitination as a crucial PTM to induce protein degradation, activation, and nuclear entry, finding host proteins that can regulate ubiquitination of MERS-CoV protein implies the discovery of a potential antiviral factor. Our previous study identified a host E3 ligase, HUWE1, that specifically induced MERS-CoV ORF3 ubiquitination and its degradation through the ubiquitin proteasome system, resulting in the diminished ability of ORF3 to induce apoptosis (55). In this work, we identified another E3 ligase that induced ORF4b degradation through the ubiquitin proteasome system. ORF3 protein can induce apoptosis, while the ORF4b protein can inhibit the innate immune pathway, both of which were associated with the pathogenicity of MERS-CoV. This gives us the insight that among the viral proteins of MERS-CoV, more than one of them can be recognized and degraded by the host proteasome. It is worthwhile to investigate whether the other MERS-CoV proteins could also undergo ubiquitination and degradation. Also, our work suggests that the host can use multiple E3 ligases to target and degrade different viral proteins. Such a discovery could provide useful information in identifying novel targets for therapy to reduce the pathogenicity of MERS-CoV.

In addition to E3 ligases, there was a class of deubiquitinases (DUBs) that removed ubiquitin from substrate proteins, thereby attenuating their ubiquitination. Interestingly, in the bioinformatics analysis, we found that in addition to UBR5, ORF4b may also interact with USP7, a DUB that could release ubiquitin molecules from the substrate protein, thus stabilizing the target protein (56, 57). It is likely that ORF4b can counteract its degradation with the help of USP7, thus reinforcing the immune escape ability of MERS-CoV by attenuating the UBR5-mediated ORF4b degradation. Once we identify the DUB, we can screen or design compounds with inhibition of DUB activity to enhance the degradation of ORF4b. In addition, to better determine the effect of ORF4b ubiquitination on MERS-CoV replication, the construction of a ubiquitination-resistant ORF4b mutation is necessary. The use of reverse genetics to build mutant strain remains to be explored in future studies.

In summary, our work identified a key E3 ligase, UBR5, that transferred ubiquitin to ORF4b to form polyubiquitin chains at lysine 36 via directly binding to the HECT domain of UBR5, resulting in the recognition and degradation of ORF4b by the proteasome. This proteolytic ubiquitination then inhibited the subsequent ORF4b-mediated suppression of IRF3 signaling. Our discoveries of the regulation of ORF4b by the ubiquitin proteasome system and the anti-MERS-CoV function of UBR5 enrich our current understanding of host resistance to MERS-CoV. Our findings provide a promising basis for the development of candidate therapy with ORF4b-UBR5 interaction as the novel molecular target.

## MATERIALS AND METHODS

### Cell lines.

Primary human airway epithelial cells were purchased from Procell (CP-H016) and cultured in the specific medium CM-H016. HEK293T and BEAS-2B cells were maintained in Dulbecco’s modified Eagle’s medium (DMEM) (Gibco), Calu3 cells were cultured in MEM (Gibco), and the other cells, including A549, HCT116, HT29, NCM460, MCF7, Hela and Huh7 cells, were grown in RPMI 1640 (Gibco). All cell lines were supplemented with 10% fetal bovine serum (FBS) (Zeta Life), penicillin (100 U/mL), and streptomycin (100 μg/mL) and cultured at 37°C with 5% CO_2_.

### Virus.

The ChinaGD01 strain of MERS-CoV was kindly provided by Jincun Zhao (State Key Laboratory of Respiratory Disease, National Clinical Research Center for Respiratory Disease). All of the experiments related to live virus were performed in biosafety laboratory 3 platform.

### Plasmids and antibodies.

The HA-tagged MERS-CoV viral genes were acknowledged to Wenjie Tan, Chinese Center for Disease Control and Prevention. Flag-tagged UBR5, the C2768A mutant, and MYC-tagged different domains of UBR5 were kindly provided by Guoqiang Xu, Soochow University. Green fluorescent protein (GFP)-tagged UBR5 was kindly provided by Yong Tae Kwon, Seoul National University. All of the other genes with different tags or mutants were subcloned into pCAGGS vector. Lentiviral shRNA clones targeting human UBR5 and the nontargeting control plasmid were purchased from Tsingke. The following antibodies were used in immunoblotting: anti-Flag (Sigma), anti-HA (CST), anti-V5 (CST), anti-GFP (CST), anti-GST (CST), anti-ubiquitin (LifeSensors), anti-UBR5 (CST), anti-IRF3 (Abclonal), anti-p-IRF3 (Abclonal), anti-NF-κB p65 (Abclonal), anti-β-actin (Sigma), and anti-histone (CST).

### Dual-luciferase reporter gene assay.

The firefly luciferase reporter plasmids containing IFN-β promoter, IRF3 responsive element, or NF-κΒ responsive element were cotransfected into HEK293T cells with *Renilla* luciferase expression vectors at a ratio of 10:1 (firefly-*Renilla*). Twenty-four hours after transfection, the cells were infected with Sendai virus (SeV) (100 HAU/mL) for 12 h, and the medium was removed. After being washed with phosphate-buffered saline (PBS) for three times, the cells were lysed and used to measure luciferase activity by the indicated protocol (dual-luciferase reporter gene assay kit, 11402ES60; Yeasen). The relative firefly luciferase activity levels were normalized to the luciferase activity of the *Renilla* control plasmid after subtracting the value of the blank control group. Data represent the mean ± standard deviation (SD) from three independent experiments.

### Real-time quantitative PCR.

Total RNA was isolated from the cells using TRIzol (Invitrogen), and 1 μg RNA was reverse transcribed to synthesize cDNA using the RevertAid first strand cDNA synthesis kit (Thermo) according to the manufacturer’s protocol. Quantitative reverse transcription-PCR (qRT-PCR) was performed using SYBR green master mix (Monad), and the threshold cycle (*C_T_*) values of the samples were measured with a LightCycler 96 (Roche). The relative level of target gene was calculated as two power values of △*C_T_* (*C_T_* of target cDNA − *C_T_* of housekeeping gene β-*actin*). All the primers used are listed in Table 1.

**TABLE 1:** Primers for qRT-PCR

Gene	Sequence of:	
	Forward primer	Reverse primer
Human *IFNβ*	5′-AGGACAGGATGAACTTTGAC-3′	5′-TGATAGACATTAGCCAGGAG-3′
Human *ISG56*	5′-TCTCAGAGGAGCCTGGCTAA-3′	5′-TGACATCTCAATTGCTCCAG-3′
Human *ISG54*	5′-ACGCATTTGAGGTCATCAGGGTG-3′	5′-CCAGTCGAGGTTATTTGGATTTGGTT-3′
Human *ISG15*	5′-GCGAACTCATCTTTGCCAGTA-3′	5′-AGCATCTTCACCGTCAGGTC-3′
Human *CCL5*	5′-CCTGCTGCTTTGCCTACATTGC-3′	5′-ACACACTTGGCGGTTCTTTCGG-3′
Human *CXCL10*	5′-CACCATGAATCAAACTGCGA-3′	5′-GCTGATGCAGGTACAGCGT-3′
Human β-*Actin*	5′-GTTGTCGACGACGAGCG-3′	5′-GCACAGAGCCTCGCCTT-3′

### Immunoprecipitation.

Total proteins were extracted with cell lysis buffer (50 mM Tris-HCl [pH 7.4], 150 mM NaCl, 1% NP-40, 5 mM EDTA, 5% glycerol) supplemented with protease inhibitor cocktail (Bimake). The whole-cell lysates were transferred to a new tube and incubated with anti-Flag agarose beads at 4°C overnight after centrifugation at 12,000 rpm for 20 min. Unbound protein was washed by lysis buffer for three times. Samples were eluted by boiling at 100°C for 5 min with protein loading buffer (0.01% bromophenol blue, 0.1 M dithiothreitol [DTT], 6% glycerol, 2% SDS, 0.25 M Tris-HCl [pH 6.8]). The lysates and immunoprecipitations were detected by the indicated primary antibodies.

### GST pulldown assay.

For GST pulldown with MERS-CoV ORF4b protein, BL21(DE3) cells containing pGEX-GST-ORF4b plasmid were induced with 0.5 mM IPTG (isopropyl-β-d-thiogalactopyranoside) at 25°C for 6 h and lysed in PBS with 100 mg/mL lysozyme, 1% Triton X-100, 0.1% sarcosyl, 1 mM DTT, and protease inhibitor cocktail. The lysate was centrifuged, passed through a 0.45-μm-pore filter, and incubated with glutathione *S*-transferase (GST) agarose (AS044; Abclonal) at 4°C for 4 h. The beads were then washed with PBS containing 1% Triton X-100 and 0.1% sarcosyl. HEK293T cells were lysed in binding buffer (20 mM Tris-HCl [pH 7.5], 100 mM NaCl, 10% glycerol, and 0.5% NP-40) with protease inhibitor cocktail. Cell lysates were centrifuged, and the supernatant was incubated with agarose beads containing GST or GST-ORF4b at 4°C for 4 to 6 h. The beads were washed with binding buffer for three times and resolved with protein loading buffer. The target proteins were detected by immunoblotting.

### Immunofluorescence.

Cells were inoculated into culture dishes with preplaced coverslips, which were removed when the cells were close to 90% confluence, and washed twice with PBS. Cells were fixed with 4% paraformaldehyde for 15 min and permeabilized with 0.2% Triton X-100–PBS solution for 20 min at room temperature. Cells were then blocked with bovine serum albumin (BSA)-PBS solution for 30 min at room temperature, followed by incubation with primary antibody (1:500) for 1 h at room temperature or overnight at 4°C. PBS-Tween (PBST) rinses were performed three times, with each rinse for 5 min. The secondary antibody (1:2,000) was incubated at room temperature and protected from light for 1 h. DAPI (4′,6-diamidino-2-phenylindole) was added to the slides to stain the nucleus, and the slides were imaged with a Leica confocal microscope. The antibodies were anti-Flag (Sigma), anti-UBR5 (Proteintech), anti-mouse conjugated with Alexa Fluor 594, and anti-rabbit conjugated with Alexa Fluor 488 (Invitrogen).

### Protein half-life assay.

Lipofectamine 3000 transfection was performed when the cells in a 6-cm dish reached about 70% confluence. Four micrograms of plasmids expressing ORF4b or mutants was used in transfection as indicated. At 12 h posttransfection, the cells were digested, resuspended, and split evenly into the wells of 12-well plates. Twenty-four hours later, when the cells reached about 90% confluence, they were treated with protein synthesis inhibitor cycloheximide (CHX) (30 μg/mL) or cotreated with MG132 (20 μM), and then the cells were collected at the indicated times. The level of target protein was detected by Western blotting.

### Stable cell line generation.

HEK293T cells were transfected with the lentivirus packaging plasmids VSV-G and DR8.9 and the lentiviral shRNA plasmids. Forty-eight hours after transfection, the supernatant was harvested and filtered. HEK293T cells were infected with the lentivirus in the presence of Polybrene (6 μg/mL) with centrifugation at 1,800 rpm for 45 min at 30°C. Cells were selected 72 h postinfection with puromycin (1 to ~2 μg/mL). The knockdown efficiency of UBR5 was detected by immunoblotting with anti-UBR5 antibody. The sequences of shRNA were as follows: shUBR5 no. 1, 5′-GCTCGTCTTGATCTACTTTAT-3′; shUBR5 no. 2, 5′-TTGGAACAGGCTACTATTAAA-3′.

### *In vivo* ubiquitination assay of ORF4b.

HEK293T cells were transfected with ORF4b and ubiquitin-expressing plasmids. Cells were treated with MG132 (20 μM) for 8 h before collection and lysed in cell lysis buffer supplemented with 0.1% SDS and 10 mM DTT. The lysates were centrifuged to remove the pellets and incubated with anti-Flag agarose beads at 4°C for 4 h. Then the beads were washed three times with cell lysis buffer. The immunoprecipitated proteins were boiled in protein loading buffer and then released from the beads and analyzed by Western blotting.

### *In vitro* ORF4b ubiquitination assay.

The MERS-CoV ORF4b was cloned and expressed as GST fusion proteins. The target viral protein was expressed in BL21 bacterial cells and purified with glutathione Sepharose beads. In addition, the Flag-UBR5 and Flag-UBR5-C2768A were purified from HEK293T cells. The beads with ORF4b protein were incubated with UBR5-WT or C2768A mutant, together with 200 ng of E1 (UBE1; R&D Systems), 400 ng of E2 (UbcH5b; R&D Systems), and 2 μg of recombinant ubiquitin (R&D Systems) in reaction buffer (50 mM Tris [PH 7.4], 2 mM MgCl_2_, 4 mM ATP [Sigma]) at 37°C for 1 h. The supernatant was removed, and the reaction was terminated by adding 2× protein loading buffer. The ubiquitin chain was detected by immunoblotting.

### Nuclear and cytoplasmic protein extraction.

The cells were collected by centrifugation, and supernatant was aspirated as much as possible, followed by treatment with cell plasma protein extraction reagent A supplemented with a protease inhibitor cocktail (Bimake). The cells were vortexed vigorously at maximum speed for 5 s to completely suspend and disperse the cell precipitate. The cells were placed in an ice bath for 10 to 15 min and then treated with 10 μL of cell plasma protein extraction reagent B. The supernatant, which contained the cytoplasmic proteins, was taken after vortexing and centrifugation. The precipitate was treated with cellular nucleoprotein extraction reagent supplemented with protease inhibitor cocktail and vortexed vigorously for 15 to 30 s at 1- to 2-min intervals for a total of 30 min. After centrifugation, the supernatant was transferred to a new tube as the nuclear proteins.

### Protein purification.

For identification of the interacting proteins and ubiquitination sites of the ORF4b protein, HEK293T cells were transfected with the Flag-ORF4b-expressing plasmid or empty vector. Forty-eight hours after transfection, cells were collected and lysed with cell lysis buffer (50 mM Tris-HCl [pH 7.4], 150 mM NaCl, 1% NP-40, 5 mM EDTA, 5% glycerol) supplemented with a protease inhibitor cocktail (Bimake). Whole-cell lysates (WCLs) were sonicated, incubated at 4°C for 20 min on a rotator, and then centrifuged for 20 min. The supernatant was filtered through a 0.45-μm-pore membrane and incubated with anti-Flag beads overnight. The proteins were eluted from the beads by 0.2 M glycine-HCl buffer (pH 3.0), and neutralized with 1.0 M Tris-HCl buffer (pH 9.0). Protein concentrations were analyzed by SDS-PAGE and silver staining with BSA as a standard.

### MERS-CoV infection and detection.

An indirect immunofluorescence assay (IFA) was used to detect S protein expression in MERS-CoV-infected cells. Huh7 cells infected with the virus (MOI = 0.0025, 0.005, or 0.01) were fixed using 4% paraformaldehyde (PFA) in PBS, and then the cells were permeabilized with 0.2% Triton X-100 in PBS. Cells were incubated with rabbit anti-MERS-CoV S protein polyclonal antibody (Sino Biologicals) at room temperature for 1 h and then stained with Fluor 594-conjugated goat anti-rabbit antibody (Abcam) (1:500 diluted in PBS containing 1% BSA); the nucleus was stained with DAPI. After being washed three times with PBS, the coverslips were mounted on glass slides. Cells were examined by confocal microscopy (Zeiss).

### Statistical analysis.

All results are shown as the mean ± SD. Student's *t* test (unpaired, two-tailed) was used to compare two independent groups, while two-way analysis of variance (ANOVA) was performed for comparisons of multiple groups. All statistical analyses were performed using GraphPad Prism 7. *P* values of <0.05 were considered statistically significant. All experiments were repeated three times or more.

### Data availability.

The data sets generated during this study are included in the published article and are available from the corresponding author on reasonable request. The mass spectrometry proteomics data have been deposited in the ProteomeXchange Consortium (http://proteomecentral.proteomexchange.org) via the PRIDE repository under the data set identifier PXD032092.
