# Supplementary material for: UBR5 Acts as an Antiviral Host Factor against MERS-CoV via Promoting Ubiquitination and Degradation of ORF4b
Source: J Virol. 2022 Aug 18;96(17):e00741-22. doi: 10.1128/jvi.00741-22 (PMC9472757; doi:10.1128/jvi.00741-22)
Supplement: Supplemental file 1 — Fig. S1-S5. Download jvi.00741-22-s0001.pdf, PDF file, 4.3 MB [file jvi.00741-22-s0001.pdf]

UBR5 acts as an antiviral host factor against MERS-CoV via promoting ubiquitination and degradation of ORF4b

Yuzheng Zhou[a], Rong Zheng[a], Donglan Liu[b], Sixu Liu[a], Cyrollah Disoma[a], Shiqin Li[a], Yujie Liao[a], Zongpeng Chen[a], Ashuai Du[a], Zijun Dong[c], Yongxing Zhang[a], Pinjia Liu[a], Aroona Razzaq[a], Dingbin Chen[b], Xuan Chen[d], Xiankezi Zhong[a], Sijie Liu[a], Siyi Tao[a], Yuxin Liu[a], Lunan Xu[a], Xu Deng[d], Jiada Li[e,h], Taijiao Jiang[f], Jincun Zhao[b,g], Shanni Li[# a] and Zanxian Xia[# a,h]

[a]Department of Cell Biology, School of Life Sciences, Central South University, Changsha 410013, China.

[b]State Key Laboratory of Respiratory Disease, National Clinical Research Center for Respiratory Disease, Guangzhou Institute of Respiratory Health, the First Affiliated Hospital of Guangzhou Medical University, Guangzhou 510182, China.

[c]Department of Basic Medicine, School of Medicine, Hunan Normal University, Changsha 410081, China.

[d]Xiangya School of Pharmaceutical Science, Central South University, Changsha 410013, China.

[e]Hunan International Scientific and Technological Cooperation Base of Animal Models for Human Disease, Changsha 410013, China.

[f]Center for Systems Medicine, Institute of Basic Medical Sciences, Chinese Academy of Medical Sciences & Peking Union Medical College, Beijing 100730, China.

[g]Institute of Infectious disease, Guangzhou Eighth People's Hospital of Guangzhou Medical University, Guangzhou 510182, China.

[h]Hunan Key Laboratory of Animal Models for Human Diseases, Hunan Key Laboratory of Medical Genetics & Center for Medical Genetics, School of Life Sciences, Central South University, Changsha 410013, China.

[#]Correspondence: Zanxian Xia, Email: xiazanxian@sklmg.edu.cn; Shanni Li, Email: shannili205121@csu.edu.cn.

Supplementary Figure 1

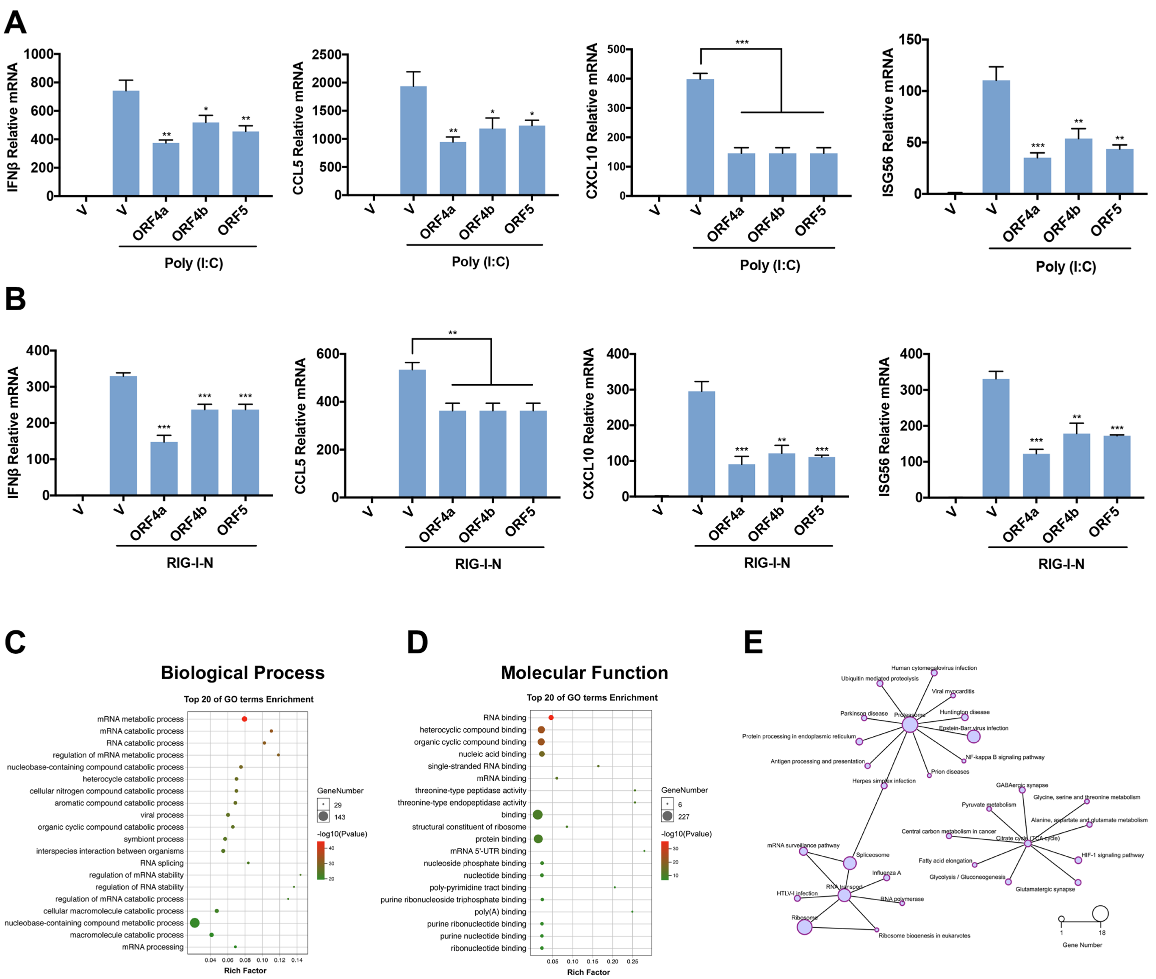

FIG S1 ORF4b is an unstable protein with dose-dependent inhibition of antiviral cytokines. **(A-B)** HEK293T cells were transfected with empty vector or plasmids expressing indicated viral protein. At 24h post transfection, cells were then transfected with Poly(I:C) (A) or RIG-I-N expressing plasmids (B) for 12h. Total RNA was extracted, reverse transcribed and analyzed by real-time PCR with primers specific for IFNβ, CCL5, CXCL10 and ISG56. Error bars indicate SD of technical triplicates. Statistical significance was calculated by unpaired, two-tailed Student's *t* test. $*p < 0.05$; $**p < 0.01$; $***p < 0.001$. **(C-D)** GO enrichment analysis for the MERS-CoV ORF4b interacting proteins, including Biological process enrichment analysis (C) and Molecular function enrichment analysis (D). **(E)**

KEGG pathways network based on the KEGG enrichment analysis.

Supplementary Figure 2

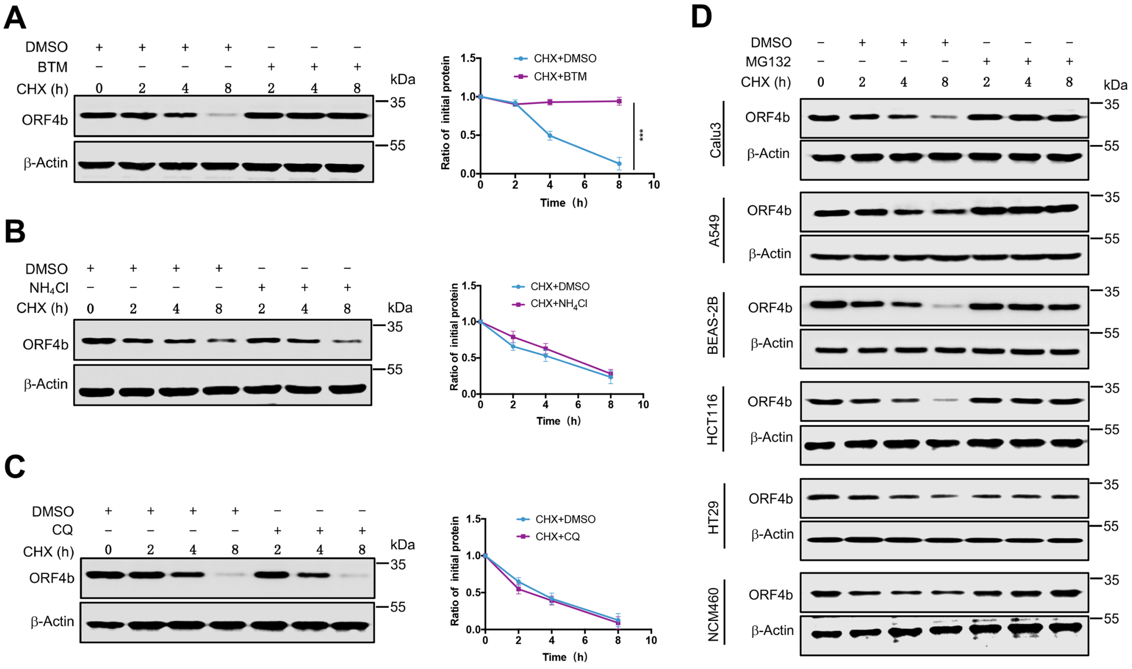

FIG S2 ORF4b is degraded by ubiquitin-proteasome system. **(A-C)** HEK293T cells transfected with ORF4b expressing plasmid were co-treated with CHX (30 μg/ml) and DMSO, or co-treated with CHX and Bortezomib (BTM , 10 μM) (A), CHX and NH_4Cl (10 mM) (B), CHX and Chloroquine (CQ, 20 μM) (C). Cells were collected at indicated time for western blotting (left). Quantification of ORF4b protein levels relative to β-actin was shown. Results were shown as mean±s.d. n=3 independent experiments. $*p < 0.05$; $**p < 0.01$; $***p < 0.001$; two-way ANOVA (right). **(D)** Plasmids expressing HA-tagged ORF4b were transfected into lung-associated cells and intestine-associated cells, including Calu3, A549, BEAS-2B, HCT116, HT29 and NCM460 cell lines, respectively. The cells were co-treated with CHX and DMSO, or co-treated with CHX and MG132 (20 μM), and then collected as indicated. The protein levels of ORF4b were detected by anti-HA antibody.

Supplementary Figure 3

A

Gene Name	Description	Peptides Ctrl-1	Peptides ORF4b-1	Peptides Ctrl-2	Peptides ORF4b-2
UBR5	HUMAN E3 ubiquitin-protein ligase UBR5	0	7	0	16
HUWE1	HUMAN E3 ubiquitin-protein ligase HUWE1	0	12	0	16

B

#	Identified peptides of UBR5 ORF4b-1	Identified peptides of UBR5 ORF4b-2	Identified peptides of HUWE1 ORF4b-1	Identified peptides of HUWE1 ORF4b-2
1	KGLLDVLPKN	KAMNQQTTLDTPQLERK	KLDNMNVSRK	KFLQFVTGTSKV
2	KLLLAIKT	KGLLDVLPKN	KVSDGGSSSTDFKM	KGNDTPLALESTNTEKE
3	RLDLLYRL	KLLLAIKT	RDMILKQ	KSILTLSHEPKV
4	KVNEEQWSLRE	RLDLLYRL	RDQSAQCTASKS	KVSDGGSSSTDFKM
5	RNAQNPSLHHPRA	KMSMTERQ	RDSAVAISGADSRG	RDGGSGNSTIIVSRS
6	RTSDSPWFLSGSETLGRL	KNTPVQSPVSLGEDLQWWPDKD	REYNLEQQRR	RDHVFEDSYRE
7	RVLLLPLERD	KTEILAVNVDSKG	RGEPVIRE	RDLSMSEEDQMMRA
8		KVNEEQWSLRE	RLLAELVR	RDSAVAISGADSRG
9		RENGADSILDLGLVDSSEKV	RSHHAASTTTAPTPAARS	REYNLEQQRR
10		RESVLRL	RSLLSILQRS	RGEEDTGQEEGGSRR
11		RIDELQVVKT	RTVLNQILRQ	RGSGTASDDEFENLRI
12		RMLVVAEQPLHAMRK	RVLDFDVKR	RLGSSGLGSASSIQAAVRQ
13		RNAQN*PSLHHPRA		RLLGPSAAADILQLSSSLPLQSRG
14		RTSDSPWFLSGSETLGRL		RQQQAATSESSQSEASVRR
15		RVLLLPLERD		RSAATSGAGSTTSGVVSGSLGSRE
16		RWSSGVGGSGGGSSGRS		RTVLNQILRQ

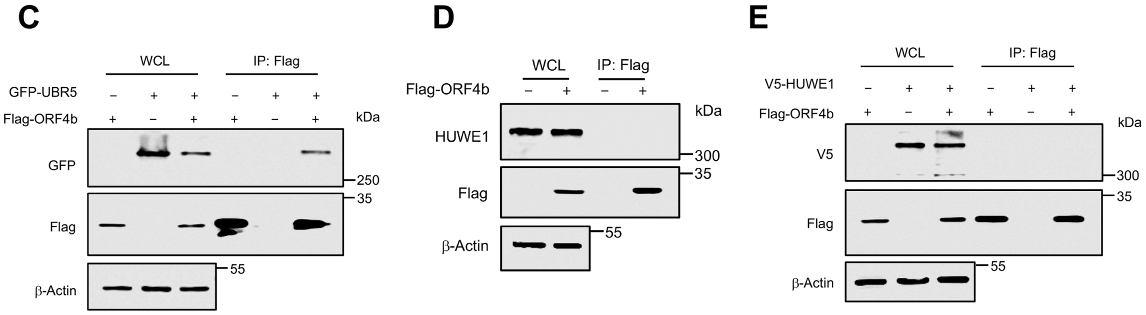

FIG S3 ORF4b interacts with UBR5. **(A)** A table showing the number of identified peptides of UBR5 and HUWE1 in different groups. **(B)** A table showing the sequences of identified unique peptides of UBR5 and HUWE1 in different groups. **(C)** GFP-UBR5 and Flag-ORF4b expressing plasmids were transfected into HEK293T cells. 48h after transfection, cells were lysed and immunoprecipitated with anti-Flag beads. The whole

cells lyses and precipitated proteins were detected with anti-GFP and anti-Flag beads by western blotting. **(D-E)** The plasmid expressing Flag-ORF4b was transfected into HEK293T cells alone (D) or co-transfected with V5-HUWE1 plasmid (E). The whole cell lyses were immunoprecipitated with anti-Flag beads and proteins were detected with indicated antibodies.

Supplementary Figure 4

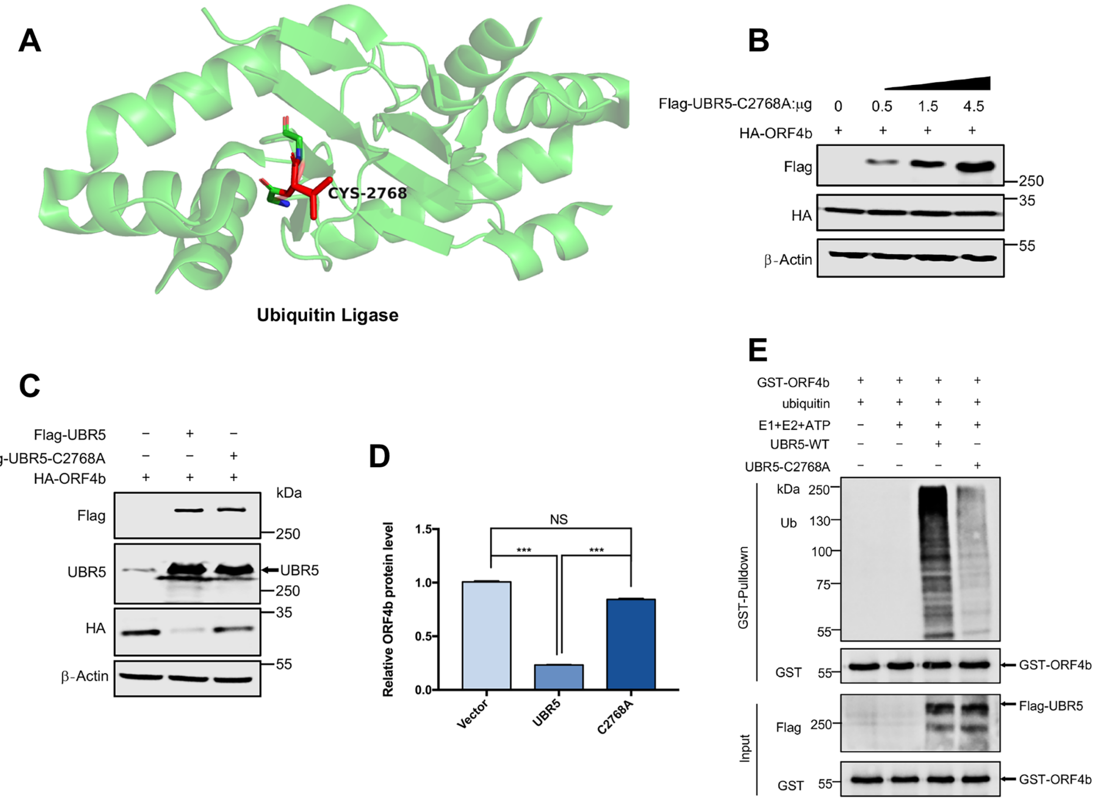

FIG S4 UBR5 mediates the ubiquitination and degradation of ORF4b. **(A)** A pdb file of UBR5 HECT domain (3pt3) was downloaded from the Protein Data Bank (PDB). Visualization of ubiquitin ligase activity site C2768 (in red) was realized with PyMOL software. **(B)** HEK293T cells transfected with ORF4b expressing plasmid were split into 12-well plate, and were then transfected with an increasing amount of plasmids containing UBR5-C2768A. The cells were collected at 48h post transfection to analyze the protein

level of ORF4b by western blotting. **(C-D)** HEK293T cells were transfected with ORF4b together with UBR5-WT , or UBR5-C2768A with deletion of E3 ligase activity. 48h after transfection, cells were collected to test ORF4b protein level (C). Quantification of ORF4b protein levels relative to β-actin was shown as mean±s.d. n=3 independent experiments. $***p < 0.001$; NS, not significant; Student's *t*-test (D). **(E)** *In vitro* ubiquitination assay of ORF4b. UBR5-WT or UBR5-C2768A were incubated with GST-ORF4b in a reaction buffer containing ATP, ubiquitin, E1 (UBE1) and E2 (UbcH5b). The ubiquitination levels were detected by immunoblotting with anti-Ub antibody.

Supplementary Figure 5

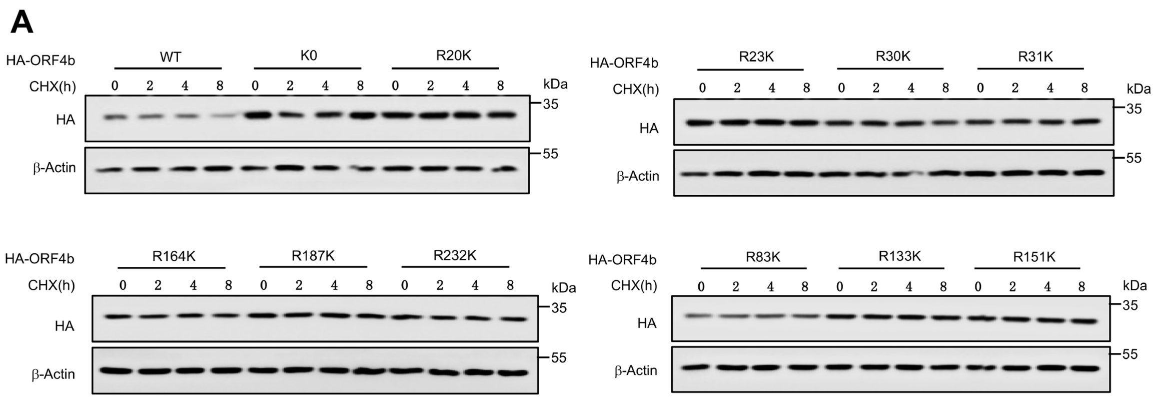

B

Location	Nucleotide locus	30 36 42
Saudi Arabia	MN120514	KKLRYVKRRFSLLRPEDL
South Korea	MK129253	KKLRYVKRRFSLLRPEDL
Egypt	MK967708	KKLRYVKRRFSLLRPEDL
Kenya	MH734115	KKLRYVKRRFSLLRPEDL
Oman	KY673148	KKLRYVKRRFSLLRPEDL
China	KT036372	KKLRYVKRRFSLLRPEDL
United Arab Emirates	KX108945	KKLRYVKRRFSLLRPEDL
Jordan	MW086537	KKLRYVKRRFSLLRPEDL

FIG S5 Ubiquitination-resistant mutant restores the stability of ORF4b. **(A)** HEK293T cells transfected with indicated plasmids were split into 12-well plates evenly. The cells

were treated with CHX (30 μg/ml) and collected at indicated time to detected the protein level of ORF4b and mutants. **(B)** The ORF4b amino acid sequences of various MERS-CoV strains from different regions were downloaded from NCBI and compared.
